# Multienvironment multilocus association mapping reveals genomic regions for drought-related traits in tropical maize

**DOI:** 10.1093/g3journal/jkag220

**Published:** 2026-08-10

**Authors:** Carina de Oliveira Anoni, Kaio Olímpio das Graças Dias, Martin Boer, Marcos Malosetti, Lauro José Moreira Guimarães, Paulo Evaristo de Oliveira Guimarães, Marcos José Andrade Viana, Maurício de Alvarenga Mudadu, Edson Alves Bastos, Milton José Cardoso, Roberto Willians Noda, Juliana E de C T Yassitepe, Jurandir Vieira de Magalhaes, Antônio Augusto Franco Garcia, Fred van Eeuwijk, Claudia Teixeira Guimarães, Maria Marta Pastina

**Affiliations:** Departamento de Genética, Escola Superior de Agricultura Luiz de Queiroz, Universidade de São Paulo (ESALQ/USP), Av. Pádua Dias 11, Piracicaba, São Paulo 13418-900, Brazil; Departamento de Biologia Geral, Universidade Federal de Viçosa, Av. PH Rolfs s/n, Viçosa, Minas Gerais 36570-900, Brazil; Biometris, Wageningen University and Research Center, Droevendaalsesteeg 2, Wageningen 6708 PB, The Netherlands; Biometris, Wageningen University and Research Center, Droevendaalsesteeg 2, Wageningen 6708 PB, The Netherlands; BASF – Nunhems, Napoleonsweg 152, Nunhem 6083 AB, The Netherlands; Embrapa Milho e Sorgo, Rodovia MG424, km 65, Sete Lagoas, Minas Gerais 35702-098, Brazil; Embrapa Milho e Sorgo, Rodovia MG424, km 65, Sete Lagoas, Minas Gerais 35702-098, Brazil; Embrapa Milho e Sorgo, Rodovia MG424, km 65, Sete Lagoas, Minas Gerais 35702-098, Brazil; Embrapa Milho e Sorgo, Rodovia MG424, km 65, Sete Lagoas, Minas Gerais 35702-098, Brazil; Embrapa Meio Norte, Rua Duque de Caxias 5650, Teresina, Piauí 64008-780, Brazil; Embrapa Meio Norte, Rua Duque de Caxias 5650, Teresina, Piauí 64008-780, Brazil; Embrapa Milho e Sorgo, Rodovia MG424, km 65, Sete Lagoas, Minas Gerais 35702-098, Brazil; Embrapa Agricultura Digital, Av. André Tosello 209, Campinas, São Paulo 13083-886, Brazil; Embrapa Milho e Sorgo, Rodovia MG424, km 65, Sete Lagoas, Minas Gerais 35702-098, Brazil; Departamento de Genética, Escola Superior de Agricultura Luiz de Queiroz, Universidade de São Paulo (ESALQ/USP), Av. Pádua Dias 11, Piracicaba, São Paulo 13418-900, Brazil; Biometris, Wageningen University and Research Center, Droevendaalsesteeg 2, Wageningen 6708 PB, The Netherlands; Embrapa Milho e Sorgo, Rodovia MG424, km 65, Sete Lagoas, Minas Gerais 35702-098, Brazil; Embrapa Milho e Sorgo, Rodovia MG424, km 65, Sete Lagoas, Minas Gerais 35702-098, Brazil

**Keywords:** multienvironment trial analysis, water deficiency, *Zea mays*, GWAS

## Abstract

Maize grain yield is frequently constrained by water scarcity, particularly in tropical regions characterized by irregular rainfall patterns. Dissecting the genetic basis of drought-related traits remains challenging because their expression is strongly influenced by environmental conditions. In this study, we applied a multienvironment multilocus genome-wide association study to identify genomic regions associated with drought-related traits in tropical maize. The association panel comprised 190 inbred lines from the Embrapa breeding program, which were genotyped-by-sequencing with 500,108 single nucleotide polymorphisms (SNPs) and crossed with 2 tester lines. Phenotypic data corresponded to the performance of the testcross hybrids, divided into Dent and Flint heterotic groups, evaluated across 2 yr at 2 locations in Brazil under well-watered and water-stressed conditions. Traits analyzed included grain yield, anthesis-silking interval, female and male flowering time, and plant and ear height. Drought stress reduced grain yield by approximately 50% and increased the anthesis-silking interval by about 2 d. A total of 179 significant SNP–trait associations were detected, of which 166 showed significant SNP-by-environment interaction effects, while 13 displayed stable effects across environments. Several associations were detected specifically under water-stressed conditions, highlighting genomic regions potentially involved in drought adaptation. Functional annotation revealed candidate genes previously implicated in abiotic stress responses, including *ZmTIP1*, which encodes an S-acyltransferase regulating root hair development and drought tolerance. Among the novel candidate genes, GRMZM2G159125, encoding a phospholipase D, emerged as a particularly promising candidate due to its strong association with grain yield and its role in membrane lipid signaling pathways related to stress responses. Although a few associations overlapped genomic regions previously reported for drought tolerance in maize, most loci represent potentially novel genetic factors that may contribute to improving drought resilience in tropical maize breeding programs.

## Introduction

Drought is one of the most severe abiotic stresses affecting maize (*Zea mays* L.), often causing substantial reductions in grain yield (GY). This constraint is particularly critical in tropical and subtropical regions, where maize is a staple crop and plays a central role in food security. In these regions, crop production is often exposed to irregular rainfall patterns, high temperatures, and increasing climate variability, which exacerbate the frequency and intensity of water deficit events. As a result, achieving stable and sustainable maize yields under adverse environmental conditions remains a major challenge for agricultural systems (Bolanos and Edmeades 1996; Ribaut et al. 2009; Almeida et al. 2013; Cooper et al. 2014). Moreover, projections of future climate scenarios indicate that drought episodes are likely to become more frequent and severe in many maize-growing regions worldwide. Consequently, the development of maize cultivars with improved water-use efficiency and enhanced drought tolerance has become a major priority for breeding programs targeting climate-vulnerable agroecosystems and aiming to ensure yield stability under water-limited conditions (Cooper and Messina 2023; Messina et al. 2023).

Drought tolerance is a complex quantitative trait controlled by multiple genes involved in diverse physiological, developmental, and metabolic pathways (Rengel et al. 2012; Tian et al. 2023). Its phenotypic expression is strongly influenced by environmental factors, including the timing, intensity, and duration of water deficit (Bruce et al. 2002). Consequently, genotype-by-environment (G × E) interactions play a major role in determining plant performance, often leading to substantial changes in the relative ranking of genotypes across locations, seasons, and management conditions (van Eeuwijk et al. 2007; Cooper and Messina 2023). These interactions complicate both the genetic dissection of drought-related traits and the identification of stable loci controlling adaptation to water-limited environments. Therefore, accurately modeling G × E effects is essential for improving the efficiency of selection in breeding programs and for understanding the genetic architecture of drought-related traits in maize (Cooper et al. 2014).

Biparental QTL mapping has been widely used to identify genomic regions associated with drought-related traits in maize (Ribaut et al. 1996, 1997; Tuberosa et al. 2002; Lu et al. 2010; Almeida et al. 2013, 2014; Malosetti et al. 2013; Ndlovu et al. 2024). This approach provides high statistical power and reduced confounding from population structure, but it is limited by low mapping resolution and restricted allelic diversity, since it captures only the allelic variations segregating between 2 parental lines. In contrast, genome-wide association study (GWAS) exploits historical recombination present in diverse germplasm panels, allowing higher-resolution detection of marker–trait associations and the identification of multiple alleles segregating within breeding populations (Hao et al. 2011; Liu et al. 2013; Xue et al. 2013; Farfan et al. 2015; Millet et al. 2016; Wallace et al. 2016; Khan et al. 2022; Chen et al. 2023; Tian et al. 2023). This increased resolution makes GWAS particularly suitable for identifying candidate genes and refining genomic regions underlying complex traits. However, GWAS can be affected by population structure and relatedness among individuals, which may lead to spurious associations if not properly controlled. To mitigate this limitation, mixed linear models incorporating kinship matrices and population structure covariates are widely used to control for confounding effects and reduce false-positive associations (Yu and Buckler 2006; Zhou and Stephens 2012).

Many GWAS for drought tolerance in maize rely on multienvironment trials (METs), which enable the evaluation of complex quantitative traits across contrasting environmental conditions and facilitate the characterization of G × E interactions (Hao et al. 2011; Xue et al. 2013; Thirunavukkarasu et al. 2014; Farfan et al. 2015; Millet et al. 2016; Wallace et al. 2016; Cooper and Messina 2023). Because drought-related traits are highly dependent on environmental conditions, MET datasets provide a valuable framework for identifying genomic regions whose effects vary across environments. In this context, analytical strategies based on 2-stage mixed-model approaches have become widely used for handling large and often unbalanced MET datasets. In the first stage, adjusted means of genotypes are estimated separately within each environment while accounting for experimental design and environmental effects. These adjusted means are subsequently used in the second stage for genome-wide association analyses. This strategy substantially reduces computational demands while maintaining statistical power to detect marker–trait associations and G × E interactions, thereby enabling the identification of loci with both stable and environment-specific effects (Smith et al. 2001; Piepho et al. 2012).

Although several GWAS have investigated drought tolerance in maize across multiple environments, many analyses were focused on single-environment models, or traits have been evaluated independently across environments. Such approaches may fail to capture G × E interactions and the environment-specific nature of marker effects. As highlighted by Boer et al. (2007), beyond detecting genomic regions associated with complex traits, it is equally essential to characterize their stability and environmental specificity across different conditions. Distinguishing single nucleotide polymorphisms (SNPs) with main from environment-specific effects is therefore critical for accurately understanding the genetic architecture of traits whose expression is strongly influenced by environmental variation. For environment-dependent traits, such as drought tolerance, integrating G × E modeling into GWAS frameworks improves the resolution and reliability of SNP–trait associations and facilitates the identification of genomic regions with differential expression across environmental conditions, which is critical for their effective application in marker-assisted selection (MAS).

In this study, we address this gap by applying a multienvironment multilocus genome-wide association study (MEML-GWAS) to identify SNP–trait associations underlying drought-related traits in tropical maize breeding germplasm. MEML-GWAS was performed using SNP genotypes of inbred lines, whereas phenotypic data were derived from the performance of their testcross hybrids obtained by crossing lines with 1 or both Dent and Flint testers. By jointly modeling SNP main effects and SNP-by-environment (SNP × E) interactions across multiple environments, this approach enabled the detection of loci with both stable and environment-specific effects. The results provided new insights into the genetic architecture of drought-related traits in tropical maize and identified genomic regions with potential value for MAS and breeding strategies for improving drought resilience.

## Material and methods

### Plant material and experimental design

The association mapping panel consisted of 190 maize inbred lines from the Embrapa maize breeding program. These lines were genotyped via genotyping-by-sequencing (GBS) and crossed with 2 tester lines, L3 and L228-3, representing the Flint and Dent heterotic groups, respectively. Association analyses therefore relied on the genotypic information of the inbred lines combined with the phenotypic performance of their corresponding testcross hybrids. Among the 190 lines, 120 were crossed with both testers, 51 exclusively with the Dent tester, and 19 exclusively with the Flint tester, resulting in a total of 310 testcross hybrids, which were evaluated in field trials across multiple environments.

To facilitate field evaluation, the testcross hybrids were divided into 6 sets according to the tester used. Sets 1 to 3 comprised hybrids derived from the Flint tester (L3), with 61, 61, and 14 hybrids, respectively. Sets 4 to 6 comprised hybrids derived from the Dent tester (L228-3), with 80, 77, and 15 hybrids, respectively. In each set, 4 commercial maize cultivars were included as common checks. Field experiments followed a randomized complete block design. Although the hybrids within each set remained the same across trials, hybrids and checks were randomly assigned to plots within each replicate, and this allocation varied among replicates and trials. The number of replicates ranged from 1 to 3, depending on location, year, and water condition (Supplementary Table 1).

The 310 testcross hybrids were evaluated under both well-watered (WW) and water-stressed (WS) conditions at 2 locations in Brazil: Janaúba, State of Minas Gerais (dark red latosol), and Teresina, State of Piauí (red–yellow argisol), during the 2010 and 2011 growing seasons. Each plot consisted of a single row, 3.6 m long in Janaúba and 4.0 m in Teresina, with 4 plants per meter and row spacing of 0.8 m in Janaúba and 0.5 m in Teresina. Each combination of location, year, and water regime was considered as a distinct environment, resulting in a total of 8 environments. Water stress was imposed by withholding irrigation approximately 45 d after planting until harvest, targeting the flowering and grain-filling stages. Under WW conditions, irrigation was scheduled according to maize evapotranspiration estimated using the Penman–Monteith equation and appropriate crop coefficients until physiological maturity. Sprinkler irrigation was used at both locations, and all other agronomic practices followed standard maize management recommendations. Detailed environmental information for each trial is provided in Supplementary Table 1. Climatic data—including daily maximum and minimum temperature and rainfall—for these field trials were previously reported by Dias et al. (2018b). Briefly, rainfall was negligible throughout the crop cycle at Janaúba in both years, whereas at Teresina, sporadic rainfall events occurred even after the onset of the water-deficit period (see Fig. 1b in Dias et al. 2018b).

**Fig. 1.**
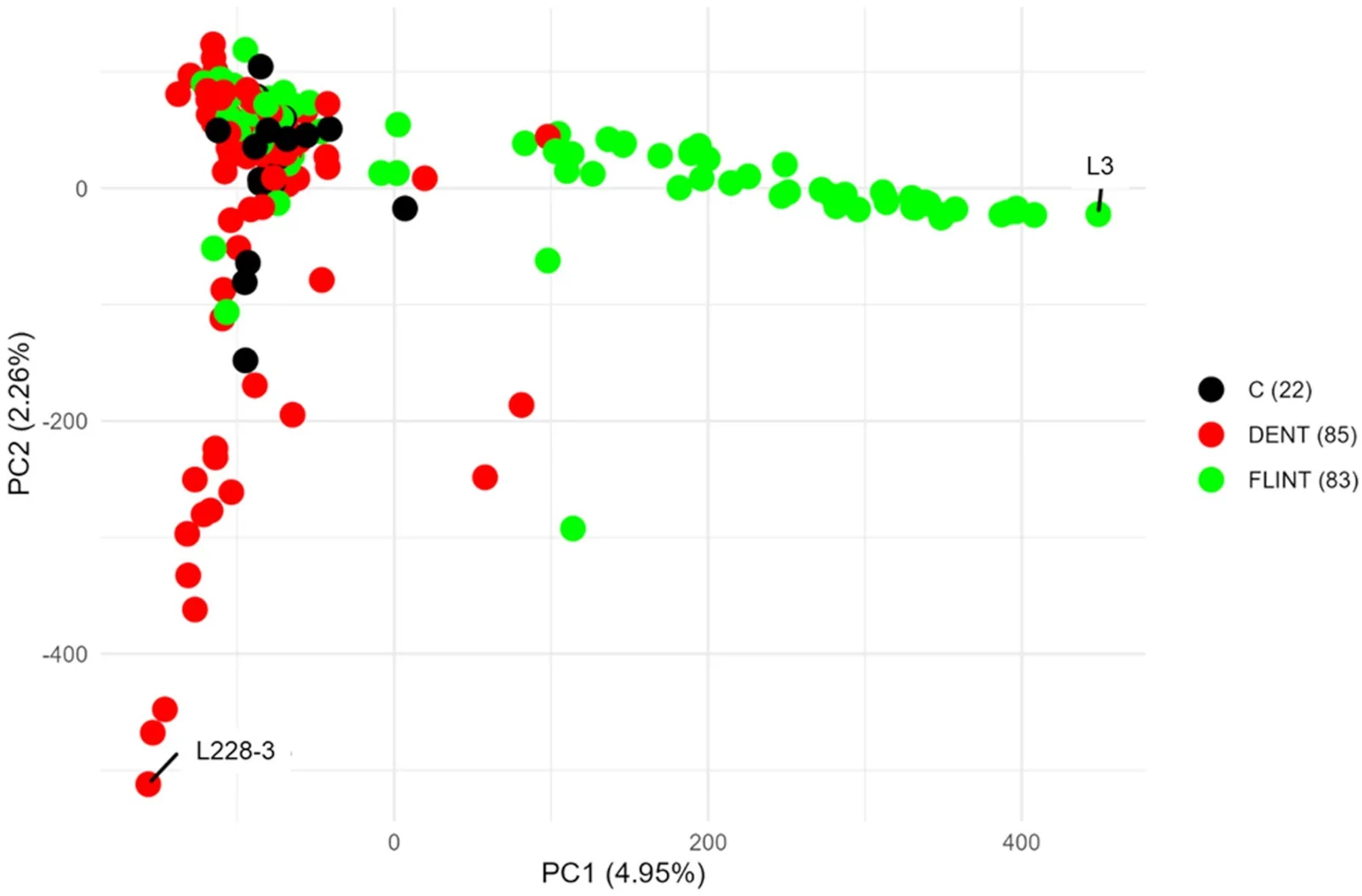
PCA using 190 maize inbred lines from the Embrapa, including Dent, Flint, and group C, which included lines with similar combining abilities when crossed with testers.

The phenotypic traits evaluated were GY, female flowering time (FF), male flowering time (MF), anthesis-silking interval (ASI), plant height (PH), and ear height (EH). GY was measured as the total grain weight per plot, adjusted to 13% moisture, and expressed in kilograms per hectare (kg ha^−1^). FF and MF were recorded as the number of days from sowing until 50% of the plants in the plot exhibited silking and pollen shedding, respectively. ASI was calculated as the difference, in days, between MF and FF. PH was measured as the distance from the soil surface to the tassel insertion (in centimeter) in 5 representative plants per plot, and EH corresponded to the distance from the soil surface to the insertion of the first ear (in centimeter) in the same 5 plants. PH and EH were not recorded in Teresina during the 2011 WW trial.

### Phenotypic data analyses of multienvironment trials

Phenotypic data were analyzed using mixed linear models in a 2-stage approach as proposed by Smith et al. (2001), which is commonly used for METs. In the first stage, single-environment analyses were performed separately for each trait using the following model (random effects are underlined):


$${\underline{Y}}_{ijk(l)}=\mu +T_{l}+{\underline{S}}_{\;j(l)}+{\underline{B}}_{k(\;jl)}+G_{i(l)}+{\underline{\varepsilon }}_{ijk(l)}$$

where ${\underline{Y}}_{ijk(l)}$ is the phenotypic observation of the *i*^th^ genotype (i=1,⋯,n) evaluated in experiment *j* (j=1,⋯,6), block *k* ($k=1,\cdots ,b_{j}$), and tester *l* (l=1,2); *μ* is the overall mean; $T_{l}$ is the effect of the *l*^th^ tester; ${\underline{S}}_{\;j(l)}$ is the effect of the *j*^th^ experiment nested within tester *l*; ${\underline{B}}_{k(\;jl)}$ is the effect of the *k*^th^ block within experiment *j* and tester *l*; $G_{i(l)}$ is the effect of the *i*^th^ genotype nested within tester *l*; and ${\underline{\varepsilon }}_{ijk(l)}$ is a residual term. Genotypes were classified into 2 groups: $n=n_{L}+n_{C}$, where $n_{L}$ is the number of inbred lines ($i=1,\cdots ,n_{L}$), and $n_{C}$ is the number of common checks ($i=n_{l}+1,\cdots ,n_{L}+n_{C}$). Accordingly, the genotypic effect $G_{i(l)}$ was modeled as:


$$G_{i(l)}=\{\begin{array}{cc} L_{i(l)}, & i=1,\cdots ,n_{L}\\ C_{i}, & i=n_{l}+1,\cdots ,n_{L}+n_{C} \end{array}$$

where $L_{i(l)}$ and $C_{i}$ represent the fixed effects of the *i*^th^ inbred line and common check, respectively. Because genotypic effects were nested within testers, adjusted means were estimated separately for each tester. Consequently, inbred lines crossed with both testers generated 2 distinct adjusted means (1 per tester), whereas those evaluated with only 1 tester produced only 1 adjusted mean. These adjusted means were subsequently used in the second stage of the analysis, in which a multi-environment association mapping was performed separately for each trait.

Genetic parameters were estimated separately for each environment by considering $G_{i(l)}$ as a random effect. Generalized heritability ($H^{2}$) was calculated following Cullis et al. (2006):


$$H^{2}=1-\frac{VBLUP}{2\sigma_{g}^{2}}$$

where *VBLUP* is the average pairwise prediction error variance of the best linear unbiased predictors (BLUP); and $\sigma_{g}^{2}$ is the estimated genotypic variance.

Phenotypic correlations between traits were estimated using a multi-environment trial analysis in which environments were grouped according to water regime: WS (J-2010-WS, J-2011-WS, T-2010-WS, and T-2011-WS) and WW (J-2010-WW, J-2011-WW, T-2010-WW, and T-2011-WW). For each trait and environment group, the following model was fitted (random effects are underlined):


$${\underline{Y}}_{ip(l)}=\mu +E_{p}+T_{l}+G_{i(l)}+{\underline{\varepsilon }}_{ip(l)}$$

where ${\underline{Y}}_{ip(l)}$ is the adjusted mean of the *i*^th^ genotype (i=1,⋯,n) in the *p*^th^ environment (p=1,⋯,4) within tester *l* (l=1,2); *μ* is the overall mean; $E_{p}$ is the effect of the *p*^th^ environment; $T_{l}$ is the effect of the *l*^th^ tester; $G_{i(l)}$ is the effect of the *i*^th^ genotype nested within tester *l*; and ${\underline{\varepsilon }}_{ip(l)}$ is the residual term, assumed to follow a MVN(0,I⊗R), where *R* is a residual variance–covariance (VCOV) matrix approximated by a diagonal structure. Adjusted means of genotypes obtained for each trait within the environment group were used to estimate phenotypic correlations using Pearson's coefficient implemented in the *corr.test* function from the psych package (Revelle 2017) in R (R Core Team 2021).

### Genotypic data

Genomic DNA was extracted from young leaves of each inbred line using the cetyltrimethylammonium bromide protocol described by Saghai-Maroof et al. (1984). DNA concentration was quantified using a Qubit 2.0 Fluorometer (Thermo Fisher Scientific, Waltham, MA, USA), and DNA integrity was evaluated by electrophoresis on 1% agarose gels prepared with Tris–acetate–EDTA buffer and stained with GelRed (Biotium, Fremont, CA, USA). Gels were visualized under UV light using a Gel Doc L-PIX imaging system (Loccus Biotecnologia, Cotia, SP, Brazil).

SNPs for the 190 inbred lines were obtained through GBS, following the protocol described by Elshire et al. (2011). Libraries were prepared using the restriction enzyme *Ape*KI, with 96 samples multiplexed per sequencing run. Sequencing was performed at the Genomics Facility of the Cornell Institute of Biotechnology (Ithaca, NY, USA). Raw sequence reads were processed using the TASSEL-GBS pipeline implemented in TASSEL v5 (Glaubitz et al. 2014). SNP discovery was performed by aligning reads to the maize reference genome B73 (AGPv3).

For each SNP, the reference allele corresponded to the nucleotide present in the B73 assembly, whereas the alternative allele represented the variant observed among the Embrapa maize inbred lines. Missing genotype calls were first imputed using the NPUTE algorithm (Roberts et al. 2007) to improve marker coverage and reduce missing data. After imputation, SNP markers were filtered to remove monomorphic loci and markers with minor allele frequency (MAF) lower than 0.05. SNP quality was assessed based on genotype call rate and imputation accuracy. The average imputation accuracy across markers was 0.96, indicating high reliability of the imputed dataset. In addition, the removal of monomorphic and low-frequency markers ensured that the retained SNPs represented reliable polymorphisms suitable for genome-wide association mapping.

### Population structure, kinship matrix, and linkage disequilibrium decay

Population structure within the association mapping panel was assessed using principal component analysis (PCA). The analysis was conducted using the full set of filtered SNP markers and implemented with the *pca* function from the *pcaMethods* package (Stacklies et al. 2007) in R. PCA was used to summarize the mains axes of genetic variation among inbred lines and to evaluate potential population stratification within the panel.

Genetic relatedness among inbred lines was estimated using a realized genomic relationship (kinship) matrix following the method proposed by VanRaden (2008). The kinship matrix was computed using the *synbreed* package (Wimmer et al. 2012) in R and was subsequently used to account for relatedness among individuals in the genome-wide association analyses.

Linkage disequilibrium (LD) between SNP markers was estimated within each chromosome by calculating pairwise squared allele-frequency correlations (*r*^2^) using the *pairwiseLD* function implemented in the *synbreed* package. Physical positions of the SNP markers were obtained from the maize reference genome B73 (AGPv3) and retrieved from the MaizeGDB database (http://www.maizegdb.org/). LD decay was evaluated by examining the relationship between pairwise *r*^2^ values and the physical distance between SNPs along each chromosome. In addition, mean LD (*r*^2^) was summarized within a sliding window of 1 Mb along each chromosome to characterize genome-wide LD patterns in relation to physical position.

### MEML association mapping

For the association mapping analyses, testcross hybrids were divided into 2 groups according to the tester used. The Flint group comprised 171 lines (51 exclusive and 120 shared) crossed with the Dent tester, whereas the Dent group included 139 lines (19 exclusive and 120 shared) crossed with the Flint tester. SNP genotypes used in the association analyses corresponded exclusively to the inbred parental lines. Therefore, genome-wide association analyses combined the genotypic information of the inbred lines with the phenotypic performance of their corresponding testcross hybrids.

To evaluate the stability and environmental sensitivity of marker–trait associations, the MEML-GWAS pipeline used a 3-step framework. First, single-environment analyses identified the best model for correcting for population structure and relatedness, followed by independent genome-wide scans across all 8 environments. Second, a joint across-environment analysis tested the marker effects simultaneously across all 8 environments rather than independently within each environment. Third, the retained marker effects were partitioned into main (stable) and environment-specific (interaction) components, classifying each SNP as either consistently expressed across environments or subject to SNP × E interactions. Each step is detailed below.

In the single-environment association analyses, 3 statistical models were evaluated to control for potential confounding effects caused by population structure and genetic relatedness among lines: (i) *Naïve model*, without correction for population structure or kinship; (ii) *PCA model*, correction for population structure through the inclusion of principal components as fixed effects; and (iii) *Kinship model*, correction for genetic relatedness using a realized genomic relationship matrix. Model performance was assessed using the genomic inflation factor (GIF), defined as the ratio between the median of the observed test statistics and the expected median under the null hypothesis. A GIF value close to 1 indicates adequate control of confounding effects, while values greater than 1 suggest inflation of the test statistics, which may arise from population stratification or relatedness among individuals. The model presenting a GIF value close to 1 was selected for the single-environment genome-wide association analyses. For each SNP, the null hypothesis assumed the absence of marker effects. Statistical significance was determined using a modified Bonferroni correction with $\alpha_{BC}=0.05$, as proposed by Matthies et al. (2014). This single-environment step served primarily to validate the correction strategy—selecting the model whose GIF was closest to unity—and produced an initial genome scan identifying significant SNP–trait associations within each environment. However, single-environment marker lists were treated as a preliminary screening step rather than the primary GWAS output.

In the second step—the primary across-environment analysis—candidate SNPs from Step 1 were jointly tested across all environments, using the previously selected model for population structure and kinship correction, and the adjusted means obtained from the phenotypic analyses. Environment-specific SNP effects were modeled as (random effects are underlined):


(1) $${\underline{y}}_{ij}=\mu +E_{j}+x_{ik}\alpha_{\;jk}+{\underline{G}}_{ij}+{\underline{\varepsilon }}_{ij}$$

where ${\underline{y}}_{ij}$ is the adjusted mean of the *i*^th^ individual in the *j*^th^ environment; *μ* is the intercept; $E_{j}$ is the effect of the *j*^th^ environment; $x_{ik}$ is the genotype of the *i*^th^ individual for the *k*^th^ SNP; $\alpha_{\;jk}$ is the environment-specific effect of the *k*^th^ SNP in the *j*^th^ environment; ${\underline{G}}_{ij}$ is the genetic residual effect of the *i*^th^ individual in the *j*^th^ environment; and ${\underline{\varepsilon }}_{ij}$ is the residual term. Residuals were assumed to follow multivariate normal distribution MVN(0,R), where *R* is a J×J residual VCOV matrix, and *J* represents the number of environments. Genetic residual effects were assumed to follow MVN(0,G), where $G=G^{A}⊗G^{E}$. In this structure, $G^{A}$ corresponds to the additive genomic relationship (kinship) matrix, and $G^{E}$ represents a J×J VCOV matrix of the genotype effects across environments. The $G^{E}$ matrix was modeled using a first-order factor analytic structure, whereas the *R* matrix was specified as diagonal. This diagonal residual VCOV structure allows environment-specific residual variances to be explicitly modeled, so that environments with lower data quality (e.g. due to reduced replication) contribute proportionally less to the joint analysis without being excluded from the multienvironment network. For each marker, the null hypothesis tested the absence of SNP effects across all environments $\left(i.e.,H_{0}:\alpha_{1k}=\alpha_{2k}=\cdots =\alpha_{\;jk}=0\right)$. This model constitutes a joint, across-environment test, rather than analyzing each environment in isolation, it evaluates the evidence for a SNP effect simultaneously across all 8 environments, providing the basis for identifying markers with consistent association signals across the multienvironment network. Significant SNP effects were identified using the *QCANDIDATES* procedure implemented in Genstat v.19 (VSN International 2017), adopting a significance threshold of α=0.0001. All markers showing significant environment-specific effects were simultaneously included in a multilocus model. A backward elimination procedure was applied to retain only significant SNPs. Markers with p−value>0.001 were sequentially removed, and marker effects were re-estimated until only significant SNPs remained in the model.

In the third and final step —the stability classification step— marker–trait associations retained from Step 2 were evaluated for the presence of significant SNP × E interaction effects, by partitioning their environment-specific effects into main (stable) and interaction (environment-dependent) components:


(2) $${\underline{y}}_{ij}=\mu +E_{j}+\overset{n_{SNP}}{\underset{k=1}{\sum }}\;(x_{ik}\alpha_{k}+x_{ik}\alpha_{\;jk})+{\underline{G}}_{ij}+{\underline{\varepsilon }}_{ij}$$

where $\alpha_{k}$ is the main effect of the *k*^th^ SNP across environments and $\alpha_{\;jk}$ is the SNP × E effect of the *k*^th^ SNP in the *j*^th^ environment. The remaining terms are defined as in Equation (1). The null hypothesis tested the absence of SNP × E effects for each marker. Because the number of SNP markers entering the multi-locus model after the first selection stage was substantially reduced relative to the initial genome-wide scan, the number of statistical tests performed in this step corresponded only to the subset of candidate SNPs retained after the backward elimination procedure (typically fewer than 200 markers). This reduction allowed the use of a less conservative significance threshold, as recommended by Malosetti et al. (2008). Markers showing significant SNP × E effects had their environment-specific effects estimated, whereas markers without interaction effects were characterized only by their main effects. This classification directly distinguishes stable, broadly expressed genomic regions from those exhibiting SNP × E effects and is reported for all significant markers in Supplementary Table 5 (summary) and Supplementary Tables 6 and 7 (Dent and Flint groups, respectively). All analyses were performed using linear mixed models in Genstat v.19 (VSN International 2017), with variance components estimated by residual maximum likelihood (Patterson and Thompson 1971).

### Candidate gene identification and functional annotation

Significant SNPs identified in the MEML-GWAS were mapped to the maize reference genome B73 (AGPv3) using *Bedtools2* to identify genes located within the genomic regions surrounding each associated SNP. Gene models overlapping or located 50 kb upstream and downstream of the significant SNPs were retrieved and considered candidate genes potentially associated with the traits analyzed. Then, to provide functional context for these candidate genes, the corresponding maize transcripts (mRNAs) were searched against the maize abiotic stress co-expression clusters. These clusters were composed of maize transcripts with correlated expression (Proost et al. 2017) derived from a co-expression network constructed using 12 maize RNA-seq experiments under abiotic stress conditions, including drought, salinity, and cold, available in the PlantAnnot2 platform (https://www.machado.cnptia.embrapa.br/plantannot; Viana et al. 2021), including the functional annotation of the predicted genes/transcripts.

Functional annotation and Gene Ontology (GO) enrichment analyses of these clusters were performed using the DAVID bioinformatics resource (Sherman et al. 2022). The best Arabidopsis homolog genes, corresponding to the maize genes from the clusters, were retrieved from Phytozome v12 (Zmays-284-Ensembl-18_2010-01 annotation data) and used as input for the DAVID analyses, including the background set, composed of all Arabidopsis homolog genes to maize. Enrichment of GO categories was evaluated using Fisher's exact test, and results were reported as fold enrichment values with corresponding *p-*values. Statistical significance was assessed using Bonferroni, Benjamini–Hochberg, and false discovery rate corrections (Sherman et al. 2022).

Additionally, to determine whether genomic regions detected in this study overlapped loci previously associated with drought tolerance in maize, SNPs and genomic intervals reported in earlier studies (Almeida et al. 2013; Avramova et al. 2017; Barbosa et al. 2021; Farfan et al. 2015; Guo et al. 2020; Khan et al. 2022; Li et al. 2016, 2024; Osuman et al. 2022; Sheoran et al. 2022; Wang et al. 2016; Xue et al. 2013; Zaidi et al. 2016; Zhang et al. 2016) were compiled and compared with the genomic positions of the SNP–trait associations identified in the present study. Because previous studies were reported using different maize genome assemblies, SNP coordinates were converted to the B73 reference genome AGPv3 using the MaizeGDB genome coordinate conversion tool. When necessary, reported SNP flanking sequences were aligned to the reference genome to confirm genomic positions and ensure accurate comparison among studies.

## Results

### Phenotypic analyses

Drought stress substantially affected maize performance across the evaluated environments. On average, GY was reduced by approximately 50% under WS compared with WW conditions, confirming the severity of the imposed stress (Supplementary Table 2). In addition, drought increased the ASI by approximately 2 d, reflecting reduced reproductive synchrony under water deficit. In contrast, PH and EH did not differ significantly between WS and WW conditions, suggesting that these architectural traits were less sensitive to the drought stress imposed in this study. Residual coefficients of variations for GY were higher under WS than under WW conditions, reflecting the greater environmental heterogeneity typically observed under water-limited conditions. This pattern has been widely reported in drought experiments. The relatively high coefficients of variation observed for ASI under WW are likely related to the low mean values of this trait and the substantial high experimental error commonly associated with ASI measurements.

Generalized heritability (*H*^2^ ) estimates varied across environments and traits, with most values ranging from moderate to high (0.40 to 0.80). The highest estimate (0.80) was observed for MF under WS conditions in Janaúba in 2010 (Supplementary Table 2). For GY, the primary trait of interest, heritability estimates ranged from 0.41 to 0.55 across the 4 WS environments, demonstrating the presence of substantial genetic variability to support the subsequent genome-wide association analyses. Under WW conditions in 2010, GY heritability estimates ranged from 0.59 to 0.60, whereas the 2 WW environments evaluated in 2011 showed markedly lower estimates (*H*^2^ = 0.12 for J-2011-WW and *H*^2^ = 0.00 for T-2011-WW; Supplementary Table 2), coinciding with the absence of replication in these 2 specific trials (Supplementary Table 1).

Phenotypic correlations estimated from the multienvironment analysis revealed distinct patterns among traits (Supplementary Table 3). GY showed negative correlations with flowering time (FF and MF) and with ASI, particularly under WS conditions, indicating that earlier flowering and shorter ASI tended to be associated with improved yield performance under drought. In contrast, positive correlations were observed between GY and both EH and PH under both water conditions. The FF and MF were strongly correlated in both conditions, with correlation coefficients of 0.88 and 0.82 under WW and WS conditions, respectively. Similarly, EH and PH were highly correlated, with coefficients of 0.75 and 0.80 under WW and WS, respectively.

### Genotypic analysis

A total of 846,948 SNPs were initially identified using the TASSEL-GBS pipeline. Missing genotype calls were imputed using chromosome-specific window sizes, resulting in an average imputation accuracy of 0.96. Approximately 35% of the SNPs exhibited a MAF below 5% and were therefore excluded from further analyses (Supplementary Fig. 1). After quality filtering, 500,108 SNPs markers were retained for the genome-wide association analyses. The number of SNPs per chromosome ranged from 77,032 on chromosome 1 to 35,541 on chromosome 10. A noticeable reduction in SNP density was observed in the central portions of the chromosomes, which likely correspond to pericentromeric regions characterized by low recombination rates and reduced marker coverage in maize genomes (Supplementary Fig. 2).

### Population structure, kinship matrix, and LD decay

PCA based on the filtered SNP markers revealed that the first 2 principal components accounted for 4.95% (PC1) and 2.26% (PC2) of the total genetic variability, totaling 7.21% (Fig. 1). LD varied across the genome, with pairwise $r^{2}$ values ranging from 0.014 to 0.310, estimated using a sliding window size of 1 Mb along each chromosome (Supplementary Fig. 3). High LD levels were observed in genomic regions close to centromeres, as well as at the distal region of chromosome 6 and the proximal region of chromosome 9. These chromosome regions also exhibited reduced marker density (Supplementary Fig. 1), a pattern commonly associated with pericentromeric regions characterized by low recombination rates in maize genomes. Overall, LD decayed rapidly across the genome, with $r^{2}$ values declining to 0.1 within approximately 1.0 to 2.0 kb (Supplementary Fig. 4), indicating a relatively high mapping resolution for the detection of SNP-trait associations in the subsequent genome-wide association analyses.

### MEML association mapping

To identify SNP-trait associations, 3 statistical models were compared: *Naïve*, *PCA*, and *Kinship*. The *Naïve* model showed the highest GIF, indicating insufficient control of confounding effects, such as population structure and genetic relatedness among individuals (Supplementary Table 4). Although the *PCA* and *Kinship* models produced similar GIF values, the *Kinship* model was selected for all subsequent analyses because it more effectively accounts for both population structure and genetic relationship.

Following the 3-step MEML-GWAS pipeline described above, single-environment analyses (Step 1) confirmed the Kinship model as the most appropriate correction structure (Supplementary Table 4) and provided an initial single-environment genome scan, while the joint across-environment analyses (Steps 2 and 3) identified the following significant marker–trait associations, reported below. MEML-GWAS identified 85 significant SNP–trait associations in the Dent group and 94 in the Flint group (Table 1; Fig. 2). These associations were distributed across all chromosomes and traits, except for EH, for which no significant loci were detected on chromosome 10 (Fig. 2). Both SNP main effects and SNP × E interaction effects were detected; however, approximately 93% of the associations exhibited significant SNP × E effects in both heterotic groups, indicating that most loci showed environment-dependent effects (Supplementary Table 5). The number of associated SNPs was distributed evenly between the Dent and Flint groups, with no evident bias toward specific traits or genomic regions. A complete list of all detected SNP–trait associations is provided in Supplementary Tables 6 and 7. In the Dent group, 7 SNPs showed significant main effects, indicating stable effects across environments: 1 associated with ASI (S2_48544791), 2 with MF (S2_60042464 and S10_114530689), 2 with PH (S7_107518590 and S9_137168948), and 2 with EH (S2_178745233 and S6_164997414) (Supplementary Table 6). In the Flint group, 6 SNPs exhibited significant main effects, including 2 associated with GY (S1_280448428 and S2_182837718), 1 with PH (S8_72256195), and 3 with EH (S1_53171490, S2_214431087, and S3_210507376) (Supplementary Table 7). Among these, the SNP S2_182837718 showed the largest estimated main effect for GY (857.7 kg ha^−1^) in the Flint group.

**Fig. 2.**
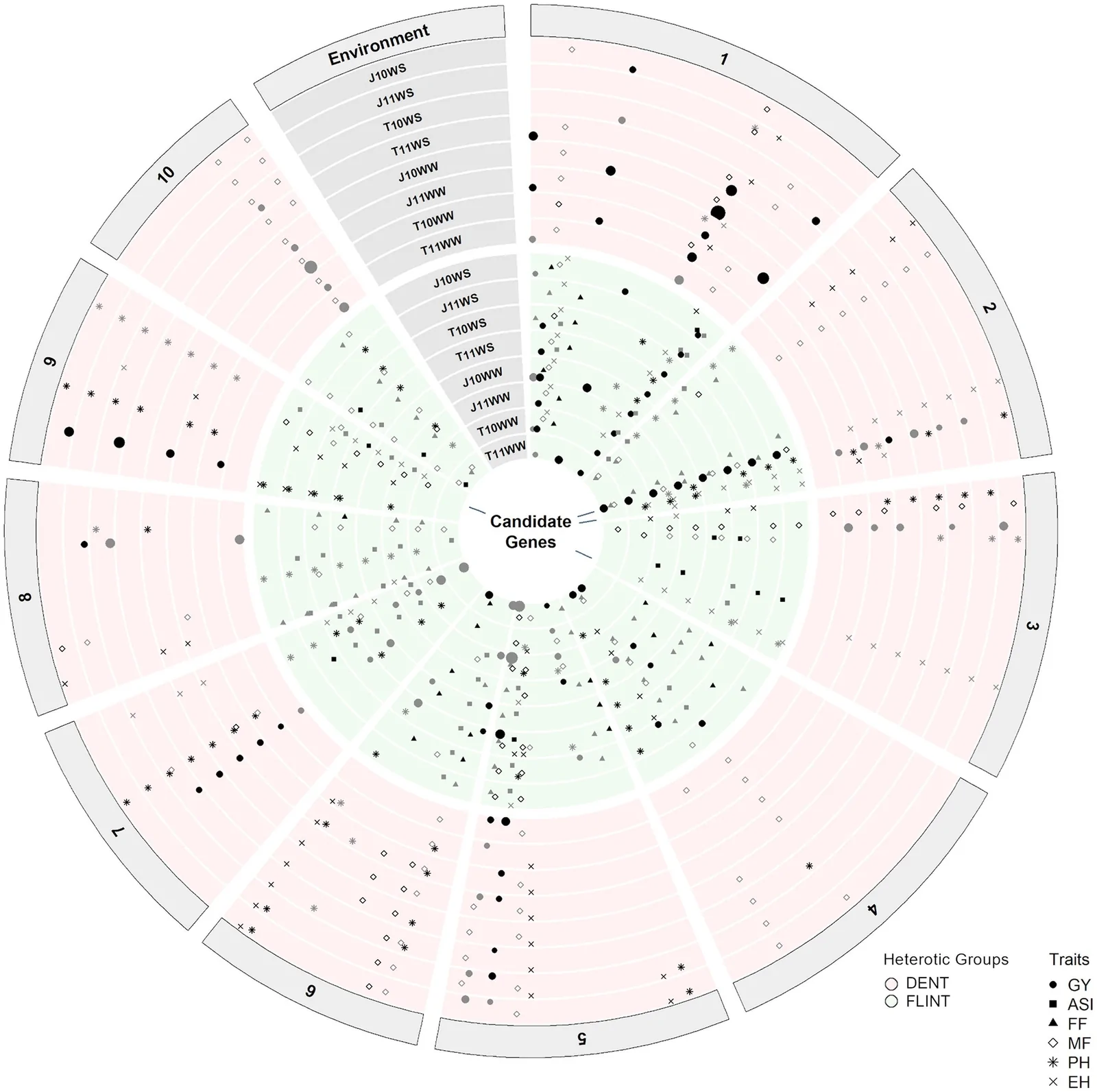
SNP–trait associations and candidate genes across maize chromosomes for the Dent and Flint heterotic groups under multienvironment trials (J10WS to T11WW). Point shapes denote distinct traits: GY, ASI, FF, MF, PH and EH. Point size is proportional to the magnitude of the main or environment-specific SNP effect.

**Table 1.** SNPs associated with traits for the Dent and Flint groups that were mapped within the predicted mRNAs and in the co-expression clusters available at PlantAnnot2 platform (https://www.machado.cnptia.embrapa.br/plantannot).

	SNPs associated with traits	SNPs mapped in mRNA	% SNPs in mRNA	SNPs mapped in the co-expression clusters^a^
Dent group	85	42	49.4	34
Flint group	94	50	53.2	46
All SNPs	500,108	248,659	49.7	

^a^More details on the expression pattern and co-expression clusters are available for selected SNPs at Supplementary Table 6.

A comparative analysis revealed limited overlap of associations between heterotic groups. Only 10 SNPs located within a physical distance of 500 kb were associated with the same genomic regions in both groups. These regions were located on chromosomes 1 (211 and 256 Mb), 2 (178 and 218 Mb), 5 (182 and 214 Mb), 6 (48 Mb), 8 (62 Mb), and 10 (4 Mb). Furthermore, few SNPs were associated with multiple traits within each heterotic group. For example, in the Dent group, SNP S8_44997484 was associated with both FF and MF (Supplementary Table 6). In the Flint group, SNPs S2_30149856 and S4_160006472 were associated with FF and MF, while S9_14465449 was associated with both PH and EH (Supplementary Table 7). These putative pleiotropic effects were consistent with the strong positive phenotypic correlations observed between FF and MF as well as between PH and EH under both WS and WW conditions (Supplementary Table 3).

Functional annotation using the PlantAnnot2 platform indicated that a substantial proportion of significant SNPs were located within coding regions: 42 of 85 SNPs in the Dent group and 50 of 94 SNPs in the Flint group. Moreover, 34 SNPs from the Dent group and 46 from the Flint group were located within co-expression clusters derived from RNA-seq experiments of maize plants exposed to abiotic stresses (Table 1). In total, 80 SNPs were associated with genes differentially expressed under drought, salinity, or cold stress, supporting the potential biological relevance of these loci. Among these, 6 SNPs with strong relevance to drought tolerance in maize are discussed in detail in the following section (Supplementary Table 8).

## Discussion

The genetic architecture of drought tolerance in maize is inherently complex, especially when phenotypic traits are evaluated across multiple environments. In this study, a genome-wide association analysis was conducted using a MEML framework to explicitly model G × E interactions affecting drought-related traits in tropical maize germplasm. By sequentially combining single-environment model validation and genome-wide scanning, joint across-environment marker detection, and explicit partitioning of main and SNP × E effects, this 3-step pipeline allowed genomic regions to be classified according to their stability across the multienvironment network, rather than relying on isolated, environment-by-environment comparisons. A total of 179 significant SNP–trait associations were detected, distributed across all chromosomes, and relatively balanced between the Dent and Flint heterotic groups. Importantly, a large proportion of these associations exhibited significant SNP × E effects, highlighting the substantial influence of environmental conditions. These results reinforce the importance of incorporating multienvironment analytical frameworks when investigating the genetic basis of drought adaptation in maize.

The modest size of the heterotic-group-specific panels used in this study (139 Dent and 171 Flint lines) is an inherent trade-off of testcross-based association mapping, where combining lines evaluated against different testers into a single panel would confound tester-specific heterotic effects with true marker–trait associations. This stratified design is consistent with previous testcross-based GWAS in maize, which similarly analyzed Dent and Flint panels separately (Revilla et al. 2016), and aligns with the hybrid development major goal of maize breeding programs. To mitigate the risk of false-positive associations arising from the modest panel sizes, several complementary safeguards were applied throughout the MEML-GWAS pipeline, including selection of the Kinship model based on GIF control, a modified Bonferroni significance threshold (Matthies et al. 2014), and a backward elimination procedure retaining only consistently significant markers in the MEML-GWAS model. Nonetheless, we acknowledge that these panel sizes are likely to reduce statistical power to detect associations of small effect or low MAF; this limitation was partially mitigated by the multienvironment phenotyping design, which increased the precision of genotype means used in the association analyses.

Genetic parameters estimated from the first-stage analyses indicated the presence of substantial genetic variability across traits and environments, as reflected by moderate to high heritability estimates ranging from 0.41 to 0.80. As expected, GY declined and ASI increased under WS conditions, patterns commonly reported in drought studies in maize (Xue et al. 2013; Dias et al. 2018a, 2018b). In contrast, PH and EH remained largely unaffected by drought, likely due to the irrigation interruption occurring after vegetative growth. Stronger phenotypic correlations between GY and phenological traits were observed under WS than under WW conditions. Notably, moderate negative correlations between GY and flowering time under WS align with the well-documented delays and floral asynchrony in drought-stressed maize (Bolaños and Edmeades 1993). The lowest heritability estimates were observed in J-2011-WW and T-2011-WW, likely reflecting the absence of replication in those trials. Consistent with this, these 2 environments also displayed disproportionately high residual variance for GY, which contributed to the overall higher absolute residual variance observed under WW relative to WS conditions. When expressed as the coefficient of variation, however, WS environments showed higher relative error variability than WW on average (26.0% vs. 17.3%, respectively), suggesting that spatial heterogeneity in soil water availability under managed drought stress amplified relative phenotypic noise, even as absolute variance remained lower due to the overall reduction in yield potential under stress. Although heritability is often lower under WS than WW conditions (Bänziger et al. 2000), this trend was not observed for several combinations of traits and environments in this study.

PCA confirmed 2 distinct genetic clusters corresponding to the well-defined Dent and Flint heterotic groups, in agreement with pedigree information. A third cluster (group C) included lines showing similar combining abilities when crossed with both Dent and Flint testers. The existence of these heterotic groups has been previously supported in a broader genetic diversity study including 1,041 maize lines from the Embrapa breeding program genotyped with 32,840 GBS-derived SNPs (Silva et al. 2020). Together, these results support the use of heterotic-group-specific association analyses in the present study. LD, measured as *r*^2^, varied across chromosomes and displayed localized peaks, including one in the distal region of chromosome 6. Interestingly, a similar LD peak in this region was reported in a panel of 240 Chinese maize lines (Liu et al. 2015), suggesting the presence of systematic genetic mechanisms underlying non-random associations in certain genomic regions. In addition, some of the regions with high LD levels observed in this study may partially correspond to pericentromeric regions, which are characterized by reduced recombination rates and extended LD blocks in maize genomes. Overall, LD decayed more rapidly in the present panel, reaching *r*^2^ = 0.1 within approximately 1.0 kb, compared with previous association studies in maize lines from the Embrapa breeding program, where LD dropped to *r*^2^ = 0.1 within approximately 10 kb (Silva et al. 2022; Ribeiro et al. 2023). Faster LD decay in tropical maize germplasm is likely driven by the higher frequency of rare alleles compared with temperate germplasm (Yan et al. 2009; Lu et al. 2011). This pattern enhances the resolution of genome-wide association analyses by increasing the likelihood of detecting significant associations near causal genes. Based on the LD decay observed in this panel, the genomic region associated with each significant SNP was defined as a 500-kb interval centered on the marker, corresponding to the approximate extent of local LD around the detected associations.

A total of 13 significant SNP–trait associations with main effects were identified: 7 in the Dent group (for ASI, MF, PH, and EH) and 6 in the Flint group (for GY, PH, and EH). Among these, the SNP S2_182837718 showed the largest estimated main effect, contributing with 857.7 kg ha^−1^ to GY in the Flint group across the 8 environments. This genomic region increased GY by approximately 18.0% under WS conditions (mean yield 4,793.2 kg ha^−1^), 9.2% under WW conditions (9,298.0 kg ha^−1^), and 12.2% across all environments (7,045.6 kg ha^−1^). Although this estimated effect size is relatively large for a single SNP, several factors may contribute to this magnitude. The detected marker may be in linkage with multiple causal variants within the region or capture indirect effects mediated by correlated traits, such as flowering time or plant architecture. In addition, the association analysis was conducted using the genotypes of inbred lines, while phenotypes corresponded to the performance of their testcross hybrids. Under this design, hybrid phenotypes reflect not only the additive genetic contribution of the parental line but also dominance interactions between alleles from the line and the tester. Because the GWAS model accounts only for the genotypes of the inbred lines, the dominance component cannot be separated from additive effects, which may lead to an overestimation of the marker effect. Therefore, further validation in independent populations will be necessary to confirm the magnitude and stability of this association.

Beyond the testcross-design considerations discussed above, the magnitude of this main effect should also be interpreted in light of the statistical safeguards embedded in the MEML-GWAS pipeline. The multi-locus framework fits multiple significant markers simultaneously as background covariates, while genetic relatedness among lines is captured by incorporating the kinship (K) matrix into the residual genetic VCOV structure, representing the polygenic background variance not attributable to the SNP under test. This strategy reduces the risk of effect-size inflation from population structure or the Beavis effect. Consistent with the heterotic-group-specific design of the analysis, an exploratory inspection of the raw phenotypic distribution of GY by allele confirmed that the direction and magnitude of the allelic difference were stable across all 8 environments within the Flint group—the panel in which this association was originally detected—whereas no comparable pattern was expected, or observed, in the Dent panel, where this SNP was not identified as GY associated. This background-specific behavior is consistent with the study's stratified, heterotic-group-specific GWAS design rather than indicative of a spurious or overestimated signal.

The SNP S2_182837718 is located within the predicted gene GRMZM2G159125, which encodes a phospholipase D (PLD). Phospholipase Dα1 has been reported to exert dual effects on plant responses to water deficits, depending on the stages and severity of drought stress (Hong et al. 2008). During early stages, *PLDα1* overexpression promotes rapid stomatal closure mediated by abscisic acid (ABA), thereby reducing transpiration and improving drought tolerance in Arabidopsis (Sang et al. 2001; Zhang et al. 2004) and in transgenic tobacco (Hong et al. 2008; Ji et al. 2018). However, under severe or prolonged drought stress, high *PLDα1* expression leads to membrane degradation and oxidative damage, ultimately reducing drought tolerance (Hong et al. 2008). In maize, the candidate gene GRMZM2G159125 exhibited a normalized transcripts per million (TPM) score of 0.05 in maize leaves under drought stress (Supplementary Table 8), indicating a low transcript abundance. Similarly, RNA-seq experiments detected low expression levels of this gene in maize under salinity and cold stresses (Supplementary Table 8). Co-expression analysis revealed that GRMZM2G159125 belongs to a cluster containing 3,161 mRNAs (zma_coexpr_cluster_0005, Supplementary Table 8), which was significantly enriched for genes involved in cell wall biogenesis and degradation processes (2.26-fold enrichment; a *P*-value of 4.24 × 10^−12^), as identified using the DAVID functional annotation platform (david.ncifcrf.gov/summary.jsp). Interestingly, this genomic region overlaps with meta-QTL MQTL2.7, previously associated with male flowering under drought stress in maize (Sheoran et al. 2022). Overall, in silico analyses suggest that GRMZM2G159125 is likely preferentially downregulated under abiotic stresses in maize, potentially as a mechanism to reduce cell wall degradation and damage.

Another main effect SNP, S3_210507376, was associated with EH in the Flint group and mapped within GRMZM2G402088, a predicted gene encoding a plant homeodomain (PHD) protein. PHD proteins participate in various aspects of plant growth and development, as well as in defense responses to pathogens and abiotic stresses (Quan et al. 2023). Recently, a member of the *PHD* gene family, *ZmVIL2*, was identified as a positive regulator of drought resistance in maize seedlings (Zhang et al. 2024). Expression of *ZmVIL2* was induced by drought, with transcript levels increasing up to 20-fold in transgenic maize plants compared to normal conditions (Zhang et al. 2024). Similarly, GRMZM2G402088 was enriched in maize leaves across 5 RNA-seq experiments under drought stress, with a normalized TPM score of up to 13.8 (Supplementary Table 8). These findings suggest that the overexpression of this candidate gene may contribute to drought tolerance mechanisms in maize. The genomic region harboring S3_210507376 (Chr3: 210,507,376) is closely linked to 2 candidate genes GRMZM2G060156 (Chr3: 211,798,637) and GRMZM2G422651 (Chr3: 211,226,056), previously associated with PH and relative ear position, respectively, under WS conditions in multi environment experiments conducted across Mexico, China, Kenya, and Thailand (Xue et al. 2013). This evidence suggests that this genomic region may harbor key genetic factors controlling PH and drought tolerance in maize. Associations with stable main effects likely reflect genomic regions whose alleles maintain consistent phenotypic effects across environments, making them highly valuable for marker-assisted breeding programs focusing on the improvement of general adaptability of maize.

In contrast, most detected associations exhibited significant SNP × E effects, representing 92% and 94% of the associations identified in the Dent and Flint groups, respectively. This pattern highlights the strong environmental modulation of drought-related traits in maize. Nevertheless, several SNPs with significant SNP × E effects showed consistent directional responses across multiple environments, indicating that their favorable alleles may still contribute positively in breeding programs. One example is the SNP S2_235397425, mapped within the predicted gene GRMZM5G805105, which encodes a C2H2 zinc-finger. This gene was identified within a co-expression cluster and was differentially expressed under drought, salinity, and cold stresses (Supplementary Table 8). Interestingly, S2_235397425 was associated with ASI in the Dent group across all 4 environments evaluated in Janaúba (2 yr × 2 water regimes), but not in Teresina. This suggests that the expression of this locus may be influenced more strongly by location-specific climatic conditions than by water availability alone. Notably, average temperatures in Teresina were 2 to 3 °C higher than in Janaúba, reinforcing that high temperatures may have contributed substantially to the observed G × E interactions. Temperature has previously been reported as a critical driver of G × E interactions in maize (Boer et al. 2007; Malosetti et al. 2013) and sorghum (Bernardino et al. 2024), often affecting flowering dynamics and ultimately contributing to yield variability under stress conditions.

Similarly, the SNP S9_95035261 was associated with ASI in the Dent group and exhibited a stable effect across 2 WW environments in Janaúba. This SNP was mapped within *ZmTIP1* (GRMZM2G087806, Chr 9: 95,033,444 to 95,044,390), a gene previously associated with drought tolerance at the seedling stage in maize (Wang et al. 2016). Functionally, *ZmTIP1* encodes an S-acyltransferase regulating root hair length and contributes to drought tolerance in transgenic Arabidopsis and maize seedlings (Zhang et al. 2020). Natural variation in the *ZmTIP1* promoter region affects gene expression levels, leading to significant differences in root hair length and survival rates of maize seedlings under severe drought stress (Zhang et al. 2020). Therefore, selecting favorable alleles at the *ZmTIP1* locus may represent promising targets for improving drought resilience in maize breeding programs.

Notably, all SNPs discussed above were located within mRNAs differentially expressed under abiotic stress experiments and clustered in co-expression networks identified through the PlantAnnot2 platform (Viana et al. 2021). This reinforces the value of PlantAnnot2 as a resource for in silico identification of candidate genes involved in plant responses to abiotic stresses. Conversely, a substantial proportion of the SNP–trait associations detected in this study did not overlap genomic regions previously reported for drought tolerance in maize. This observation suggests that tropical germplasm may harbor novel genetic variation relevant for stress adaptation. Although tropical maize has been less extensively characterized than temperate germplasm, it has been increasingly recognized as an important source of adaptive genetic diversity (Wisser et al. 2019; Tian et al. 2023). These results highlight the potential of tropical breeding germplasm to contribute new genetic resources for improving drought resilience in maize.

### Practical implications for tropical maize breeding: advantages and limitations

Beyond contributing to the understanding of the genetic architecture of drought tolerance, these results have direct implications for applied tropical maize breeding, with both opportunities and limitations for practical use. Of the 179 significant SNP–trait associations detected, only 13 (7.3%) exhibited stable main effects across environments, including S2_182837718 (GY, Flint group) and S2_48544791 (ASI, Dent group). These environment-independent loci represent the most promising candidates for direct MAS, as they could be converted into low-cost, high-throughput genotyping assays for parental line selection. However, as discussed for S2_182837718, independent validation is essential before deployment, given the potential confounding between additive and dominance effects inherent to the testcross-based design used here.

In contrast, the remaining ∼93% of associations exhibited significant SNP × E interactions, which are not well suited for direct MAS. Instead, they may be more effectively integrated into genomic selection (GS) frameworks, where GWAS-derived markers can be incorporated as weighted or fixed effects to improve the prediction models of drought-adaptive performance within target populations of environments. Previous studies have shown that incorporating known major-effect loci into GS models can increase prediction accuracy in maize (Bian and Holland 2017) and, more broadly, across complex traits (Bernardo 2014).

Despite these opportunities, several considerations temper the direct application of the identified loci in breeding programs. First, because drought tolerance is highly polygenic, the markers identified here capture only a portion of the total genetic variance and should be regarded as complementary to, rather than replacement for, GS strategies capturing the broader polygenic background. Second, because marker effects were estimated from testcrosses, their usefulness is expected to depend on the heterotic background in which they were detected and may require further validation before deployment in unrelated germplasm pools. Finally, given the strong environmental dependency of most associations, translating these findings into breeding gains will require continued evaluation in multiple environments and populations.

## Supplementary Material

jkag220_Supplementary_Data

## Data Availability

The phenotypic dataset, the high-density SNP matrix and the Genstat scripts used to perform the MEML-GWAS analyses are publicly and permanently available at the Repositório de Dados de Pesquisa da Embrapa (www.redape.dados.embrapa.br) under the DOI: https://doi.org/10.48432/FH6GFL. Supplemental material available at *G3* online.
