# Supplementary material for: Multienvironment multilocus association mapping reveals genomic regions for drought-related traits in tropical maize
Source: G3 (Bethesda). 2026 Aug 10;16(10):jkag220. doi: 10.1093/g3journal/jkag220 (PMC13643811; doi:10.1093/g3journal/jkag220)
Supplement: Supplementary file 1 [file jkag220_supplementary_data.zip › Supplemental_Material_G3-2026-406860.docx]

**Supplementary Material**

**Table S1.** Description of experimental design for trials with maize testcross hybrids derived from the association mapping panel for drought tolerance from the Embrapa maize breeding program regarding to location, year, and environment conditions (WS – water stressed; WW – Well Watered).

| Environment | J10WS | J11WS | T10WS | T11WS | J10WW | J11WW | T10WW | T11WW |
| --- | --- | --- | --- | --- | --- | --- | --- | --- |
| Location | Janaúba | Janaúba | Teresina | Teresina | Janaúba | Janaúba | Teresina | Teresina |
| Year | 2010 | 2011 | 2010 | 2011 | 2010 | 2011 | 2010 | 2011 |
| Condition | WS | WS | WS | WS | WW | WW | WW | WW |
| N.B^*^.1 | 3 | 3 | 2 | 3 | 2 | 1 | 2 | 1 |
| N.B. ^*^2 | 3 | 3 | 2 | 3 | 2 | 1 | 2 | 1 |
| N.B. ^*^3 | 2 | 2 | 2 | 2 | 2 | 1 | 2 | 1 |
| N.B. ^*^4 | 3 | 3 | 2 | 3 | 2 | 1 | 2 | 1 |
| N.B. ^*^5 | 3 | 3 | 2 | 3 | 2 | 1 | 2 | 1 |
| N.B. ^*^6 | 2 | 2 | 2 | 2 | 2 | 1 | 2 | 1 |
| Latitude | 15°48' S | 15°48' S | 05°05' S | 05°05' S | 15°48' S | 15°48' S | 05°05' S | 05°05' S |
| Longitude | 43°19' W | 43°19' W | 42°48' W | 42°48' W | 43°19' W | 43°19' W | 42°48' W | 42°48' W |
| Altitude(m) | 516 | 516 | 72 | 72 | 516 | 516 | 72 | 72 |
| Annual average Temperature (°C) | 25.1 | 24.6 | 27.9 | 27.1 | 25.1 | 24.6 | 27.9 | 27.1 |
| Max.Temperature annual average (°C) | 31.9 | 31.6 | 34.7 | 33.5 | 31.9 | 31.6 | 34.7 | 33.5 |
| Min.Temperature annual average(°C) | 19.4 | 18.9 | 22.4 | 22.3 | 19.4 | 18.9 | 22.4 | 22.3 |
| Annual rainfall (mm) | 676 | 695 | 1036 | 1591 | 676 | 695 | 1036 | 1591 |

^*^N.B. = Number of blocks at specific experiment

**Table S2.** Genetic parameters estimated for each trait of the association mapping panel for drought tolerance from the Embrapa maize breeding program across all environments: mean; mean G for groups of environments WS and WW; genotypic ($\sigma_{G}$) and residual ($\sigma_{e}$) standard deviations; residual coefficient of variation (${CV}_{e}$), not estimated for anthesis-silking interval (ASI); and generalized heritabily measure ($h^{2}$. GY: grain yield (kg.ha^-1^); ASI: anthesis-silking interval (days); FF: female flowering time (days); MF: male flowering time (days); PH: plant height (cm); EH: ear height (cm).

| Parameter | Trait | J10WS | J11WS | T10WS | T11WS |  | J10WW | J11WW | T10WW | T11WW |
| --- | --- | --- | --- | --- | --- | --- | --- | --- | --- | --- |
| Mean | GY | 4,822.96 | 4,138.71 | 4,524.30 | 5,686.74 |  | 9,003.50 | 9,819.39 | 9,886.92 | 8,482.35 |
| Mean G |  |  |  |  | 4,793.18 |  |  |  |  | 9,298.04 |
| $\sigma_{G}$ |  | 613.02 | 537.49 | 682.71 | 653.49 |  | 944.39 | 462.68 | 988.99 | 1.00 |
| $\sigma_{e}$ |  | 1,163.11 | 1,064.87 | 1,435.38 | 1,282.63 |  | 1,357.89 | 1,577.18 | 1409.78 | 1,996.48 |
| ${CV}_{e}\left( \text{\%} \right)$ |  | 24.10 | 25.70 | 31.70 | 22.60 |  | 15.10 | 16.10 | 14.30 | 23.50 |
| $H^{2}$ |  | 0.55 | 0.53 | 0.41 | 0.53 |  | 0.59 | 0.12 | 0.60 | 0.00 |
|  |  |  |  |  |  |  |  |  |  |  |
| Mean | ASI | -0.19 | 2.39 | 6.08 | 3.21 |  | 0.76 | 0.37 | 1.94 | 2.35 |
| Mean G |  |  |  |  | 2.87 |  |  |  |  | 1.36 |
| $\sigma_{G}$ |  | 0.96 | 1.07 | 0.78 | 0.42 |  | 0.76 | 0.74 | 0.22 | 0.16 |
| $\sigma_{e}$ |  | 1.31 | 1.91 | 3.58 | 0.86 |  | 0.95 | 1.12 | 0.69 | 0.58 |
| ${CV}_{e}\left( \text{\%} \right)$ |  | - | - | - | - |  | - | - | - | - |
| $H^{2}$ |  | 0.70 | 0.58 | 0.13 | 0.51 |  | 0.66 | 0.40 | 0.24 | 0.11 |
|  |  |  |  |  |  |  |  |  |  |  |
| Mean | FF | 62.47 | 70.61 | 54.85 | 56.61 |  | 65.41 | 70.09 | 52.21 | 54.44 |
| Mean G |  |  |  |  | 61.14 |  |  |  |  | 60.54 |
| $\sigma_{G}$ |  | 1.76 | 1.79 | 1.47 | 1.10 |  | 1.61 | 1.25 | 0.92 | 1.11 |
| $\sigma_{e}$ |  | 1.87 | 2.67 | 4.22 | 1.61 |  | 1.54 | 1.71 | 2.34 | 1.31 |
| ${CV}_{e}$(%) |  | 3.00 | 3.80 | 8.10 | 2.8 |  | 2.30 | 2.40 | 4.50 | 2.40 |
| $H^{2}$ |  | 0.79 | 0.66 | 0.26 | 0.67 |  | 0.76 | 0.45 | 0.33 | 0.52 |
|  |  |  |  |  |  |  |  |  |  |  |
| Mean | MF | 62.81 | 68.15 | 48.50 | 53.329 |  | 64.75 | 69.75 | 50.52 | 52.08 |
| Mean G |  |  |  |  | 52.20 |  |  |  |  | 59.27 |
| $\sigma_{G}$ |  | 1.77 | 1.59 | 1.17 | 1.00 |  | 1.33 | 1.30 | 0.87 | 1.13 |
| $\sigma_{e}$ |  | 1.33 | 1.78 | 2.48 | 1,47 |  | 1.36 | 1.60 | 2.25 | 1.21 |
| ${CV}_{e}\left( \text{\%} \right)$ |  | 2.10 | 2.50 | 5.10 | 2.80 |  | 2.10 | 2.30 | 4.50 | 2.30 |
| $H^{2}$ |  | 0.80 | 0.78 | 0.41 | 0.66 |  | 0.74 | 0.50 | 0.32 | 0.57 |
|  |  |  |  |  |  |  |  |  |  |  |
| Mean | PH | 197.23 | 224.32 | 194.18 | 210.81 |  | 184.11 | 233.12 | 218.41 |  |
| Mean G |  |  |  |  | 206.64 |  |  |  |  | 211.88 |
| $\sigma_{G}$ |  | 76.00 | 86.60 | 84.70 | 134.50 |  | 72.80 | 59.40 | 97.80 |  |
| $\sigma_{e}$ |  | 14.70 | 14.29 | 21.74 | 12.03 |  | 11.86 | 14.77 | 12.50 |  |
| ${CV}_{e}$(%) |  | 7.50 | 6.40 | 11.20 | 5.70 |  | 6.40 | 6.40 | 5.70 |  |
| $H^{2}$ |  | 0.60 | 0.65 | 0.36 | 0.79 |  | 0.61 | 0.29 | 0.65 |  |
|  |  |  |  |  |  |  |  |  |  |  |
| Mean | EH | 103.06 | 121.77 | 121.72 | 103.82 |  | 89.16 | 134.17 | 128.72 |  |
| Mean G |  |  |  |  | 112,59 |  |  |  |  | 117.35 |
| $\sigma_{G}$ |  | 6.57 | 7.30 | 8.28 | 8.26 |  | 6.74 | 8.67 | 8.87 |  |
| $\sigma_{e}$ |  | 12.23 | 10.72 | 10.85 | 8.77 |  | 9.86 | 12.04 | 10.18 |  |
| ${CV}_{e}$ (%) |  | 11.90 | 8.90 | 8.90 | 8.40 |  | 11.10 | 9.00 | 7.90 |  |
| $H^{2}$ |  | 0.56 | 0.67 | 0.63 | 0.79 |  | 0.59 | 0.44 | 0.69 |  |

**Table S3.** Average phenotypic correlation estimation based on the adjusted means obtained under water-stressed (upper diagonal) and well-watered (lower diagonal) conditions for grain yield (GY); anthesis-silking interval (ASI), female flowering time (FF), male flowering time (MF), plant heigh (PH) and ear heigh (EH) traits. Significant estimates with $p<0.01$ are highlighted in bold.

| Trait | GY | ASI | FF | MF | PH | EH |
| --- | --- | --- | --- | --- | --- | --- |
| GY |  | **-0.48** | **-0.50** | **-0.41** | **0.32** | **0.27** |
| ASI | **-0.24** |  | **0.67** | 0.10 | -0.07 | -0.13 |
| FF | **-0.33** | **0.42** |  | **0.82** | **0.16** | 0.07 |
| MF | **-0.24** | -0.03 | **0.88** |  | -0.05 | -0.03 |
| PH | **0.21** | **0.48** | 0.09 | **-0.14** |  | **0.80** |
| EH | **0.30** | **0.23** | 0.02 | -0.10 | **0.75** |  |

**Table S4.** Genomic Inflation Factors (GIF) from the Naïve, PCA, and Kinship association mapping models, obtained for each mapping group: Dent and Flint; trait: grain yield (GY), anthesis-silking interval (ASI), female flowering time (FF), male flowering time (MF), plant heigh (PH) and ear heigh (EH); and environment: J10WS, J11WS, T10WS, T11WS, J10WW, J11WW, T10WW, and T11WW.

| Model | Environment | Dent Group | | | | | |  | Flint Group | | | | | |
| --- | --- | --- | --- | --- | --- | --- | --- | --- | --- | --- | --- | --- | --- | --- |
|  |  | GY | ASI | FF | MF | PH | EH |  | GY | ASI | FF | MF | PH | EH |
| Naive | J10WS | 1.149 | 1.163 | 1.545 | 1.760 | 1.575 | 1.246 |  | 1.162 | 1.342 | 1.485 | 1.466 | 1.685 | 1.449 |
| PCA |  | 1.048 | 1.149 | 1.254 | 1.200 | 1.044 | 1.061 |  | 1.045 | 1.176 | 1.215 | 1.133 | 1.076 | 1.024 |
| Kinship |  | 1.055 | 1.049 | 1.098 | 1.124 | 1.093 | 1.047 |  | 1.011 | 1.064 | 1.070 | 1.075 | 1.069 | 1.075 |
|  |  |  |  |  |  |  |  |  |  |  |  |  |  |  |
| Naive | J11WS | 1.590 | 1.537 | 1.406 | 1.344 | 1.359 | 1.194 |  | 1.477 | 1.044 | 1.496 | 1.583 | 1.662 | 1.350 |
| PCA |  | 1.058 | 1.081 | 1.119 | 1.077 | 1.005 | 1.071 |  | 0.996 | 1.044 | 1.071 | 1.074 | 1.091 | 1.043 |
| Kinship |  | 1.168 | 1.132 | 1.217 | 1.082 | 1.055 | 1.075 |  | 1.045 | 1.026 | 1.077 | 1.067 | 1.105 | 1.053 |
|  |  |  |  |  |  |  |  |  |  |  |  |  |  |  |
| Naive | T10WS | 1.105 | 1.033 |  | 1.264 | 1.215 | 1.231 |  | 1.086 | 1.037 | 1.120 | 1.388 | 1.153 | 1.163 |
| PCA |  | 0.962 | 1.000 |  | 1.010 | 0.97 | 1.097 |  | 1.077 | 1.037 | 1.043 | 1.067 | 1.075 | 1.003 |
| Kinship |  | 0.987 | 0.992 |  | 1.024 | 1.102 | 1.087 |  | 1.042 | 1.026 | 1.042 | 1.057 | 1.003 | 1.015 |
|  |  |  |  |  |  |  |  |  |  |  |  |  |  |  |
| Naive | T11WS | 1.567 | 1.564 | 1.260 | 1.102 | 1.312 | 1.177 |  | 1.573 | 1.044 | 1.368 | 1.438 | 1.646 | 1.335 |
| PCA |  | 1.045 | 1.080 | 1.101 | 1.046 | 1.132 | 1.089 |  | 1.019 | 1.044 | 1.066 | 1.080 | 1.112 | 1.027 |
| Kinship |  | 1.126 | 1.088 | 1.093 | 1.083 | 1.129 | 1.095 |  | 1.135 | 1.036 | 1.092 | 1.111 | 1.060 | 1.036 |
|  |  |  |  |  |  |  |  |  |  |  |  |  |  |  |
| Naive | J10WW | 1.251 | 1.292 | 1.389 | 1.583 | 1.376 | 1.328 |  | 1.483 | 1.211 | 1.508 | 1.529 | 1.539 | 1.228 |
| PCA |  | 1.039 | 1.194 | 1.274 | 1.158 | 1.089 | 1.094 |  | 1.026 | 1.168 | 1.196 | 1.113 | 1.006 | 1.008 |
| Kinship |  | 1.093 | 1.064 | 1.138 | 1.196 | 1.125 | 1.098 |  | 1.019 | 1.035 | 1.049 | 1.078 | 1.051 | 1.045 |
|  |  |  |  |  |  |  |  |  |  |  |  |  |  |  |
| Naive | J11WW | 1.059 | 1.162 | 1.128 | 1.228 | 1.365 | 1.192 |  | 1.192 | 1.086 | 1.102 | 1.248 | 1.129 | 1.079 |
| PCA |  | 1.008 | 1.032 | 1.079 | 1.060 | 1.065 | 1.084 |  | 1.001 | 0.985 | 1.026 | 1.062 | 0.989 | 0.999 |
| Kinship |  | 1.072 | 1.003 | 1.068 | 1.080 | 1.078 | 1.068 |  | 1.060 | 1.075 | 1.045 | 1.076 | 1.034 | 1.034 |
|  |  |  |  |  |  |  |  |  |  |  |  |  |  |  |
| Naive | T10WW | 1.229 | 1.058 | 1.136 | 1.092 | 1.310 | 1.297 |  | 1.165 | 1.022 | 1.310 | 1.291 | 1.338 | 1.254 |
| PCA |  | 1.035 | 1.058 | 1.118 | 1.069 | 1.038 | 1.071 |  | 1.020 | 1.022 | 1.156 | 1.125 | 1.062 | 1.077 |
| Kinship |  | 1.061 | 1.030 | 1.086 | 1.080 | 1.090 | 1.085 |  | 1.043 | 1.038 | 1.083 | 1.083 | 1.073 | 1.044 |
|  |  |  |  |  |  |  |  |  |  |  |  |  |  |  |
| Naive | T11WW | 1.353 | 1.323 | 1.236 | 1.257 |  |  |  | 1.087 | 1.017 | 1.414 | 1.441 |  |  |
| PCA |  | 0.967 | 1.030 | 1.147 | 1.143 |  |  |  | 1.069 | 1.017 | 1.158 | 1.111 |  |  |
| Kinship |  | 1.016 | 1.025 | 1.165 | 1.188 |  |  |  | 1.037 | 1.031 | 1.058 | 1.040 |  |  |

**Table S5.** Number of associated SNP (Loci), SNP-by-environment (SNP x E) interactions, and the number of SNP x E by chromosome for each trait, resulting from the MEML association mapping for Dent and Flint mapping groups.

| Mapping group | Trait | Loci | Effect | | SNP x E by chromosome^*^ |
| --- | --- | --- | --- | --- | --- |
|  |  |  | Main | SNP x E |  |
| Dent | GY | 12 |  | 12 | 1^(4)^, 2^(1)^, 3^(1)^, 5^(2)^, 7^(1)^, 8^(1)^, 9^(1)^, 10^(1)^ |
|  | ASI | 19 | 1 | 18 | 1^(1)^, 2^(3)^, 3^(1)^, 4^(2)^, 5^(2)^, 6^(3)^, 7^(2)^, 8^(1)^, 9^(2)^, 10^(2)^ |
|  | FF | 14 |  | 14 | 1^(3)^, 2^(1)^, 3^(1)^, 4^(1)^, 5^(2)^, 7^(1)^, 8^(1)^, 9^(1)^, 10^(3)^ |
|  | MF | 16 | 2 | 14 | 1^(3)^, 2^(1)^, 3^(1)^, 4^(2)^, 5^(2)^, 6^(3)^, 7^(1)^, 8^(1)^, 10^(2)^ |
|  | PH | 13 | 2 | 11 | 1^(1)^, 2^(1)^, 3^(2)^, 4^(1)^, 5^(1)^, 6^(3)^, 7^(1)^, 8^(1)^, 9^(2)^ |
|  | EH | 11 | 2 | 9 | 1^(1)^, 2^(3)^, 3^(1)^, 5^(2)^, 6^(1)^, 7^(1)^, 8^(1)^, 9^(1)^ |
|  |  | **85** | **7** | **78** |  |
| Flint | GY | 13 | 2 | 11 | 1^(4)^, 2^(1)^, 4^(2)^, 5^(2)^, 6^(2)^, 7^(2)^ |
|  | ASI | 16 |  | 16 | 1^(3)^, 2^(1)^, 3^(3)^, 4^(1)^, 5^(1)^, 6^(1)^, 7^(2)^, 8^(2)^, 9^(1)^, 10^(1)^ |
|  | FF | 18 |  | 18 | 1^(3)^, 2^(2)^, 4^(4)^, 5^(3)^, 6^(2)^, 8^(2)^, 10^(1)^ |
|  | MF | 18 |  | 18 | 1^(1)^, 2^(2)^, 3^(3)^, 4^(1)^, 5^(4)^, 6^(1)^, 7^(1)^, 8^(2)^, 9^(2)^, 10^(2)^ |
|  | PH | 15 | 1 | 14 | 1^(1)^, 2^(3)^, 3^(1)^, 4^(1)^, 5^(2)^, 6^(1)^, 7^(2)^, 8^(1)^, 9^(1)^, 10^(1)^ |
|  | EH | 14 | 3 | 11 | 1^(2)^, 2^(2)^, 3^(2)^, 4^(1)^, 5^(2)^, 7^(1)^, 8^(1)^, 9^(3)^ |
|  |  | **94** | **6** | **88** |  |
| **Total** |  | **179** | **13** | **166** |  |

^*^Chromosomes with SNP x E are represented by the respective chromosome numbers. Superscribed are the numbers of loci with significant SNPE x E. GY: grain yield (kg.ha^-1^); ASI: anthesis-silking interval (days); FF: female flowering time (days); MF: male flowering time (days); PH: plant height (cm); EH: ear height (cm)

**Table S6.** Significant main and environment-specific associations detected through the MEML association mapping model for the Dent mapping group. Bold values represent significant effects considering a threshold of $2 \overline{se}$, being $\overline{se}$ the mean residual standard error. Evaluated traits were: grain yield (GY), anthesis-silking interval (ASI), female flowering time (FF), male flowering time (MF), plant heigh (PH) and ear heigh (EH). Chr: chromosome. Environment: J10WS, J11WS, T10WS, T11WS, J10WW, J11WW, T10WW, and T11WW.

| Trait | SNP^*^ | Chr | Allele^**^ | J10WS | J11WS | T10WS | T11WS | J10WW | J11WW | T10WW | T11WW | $2 \overline{se}$ |
| --- | --- | --- | --- | --- | --- | --- | --- | --- | --- | --- | --- | --- |
| GY | S1_2038013 | 1 | A/T | 287.517 | 131.795 | 286.225 | **1,110.72** | -17.231 | **745.43** | 235.328 | **-621.675** | 361.672 |
|  | S1_86369547 | 1 | G/A | **570.233** | 36.781 | **-630.925** | 318.891 | **1,297.18** | -65.62 | **717.83** | -182.218 | 425.12 |
|  | S1_211572436 | 1 | T/G | 492.527 | 633.877 | -200.162 | **1,721.93** | **2,547.04** | **832.997** | **1,372.19** | **-1,158.04** | 809.933 |
|  | S1_295519463 | 1 | A/T | 91.331 | -44.773 | **917.313** | 178.47 | 399.176 | **1,873.18** | 114.773 | 184.222 | 453.161 |
|  | S2_203181508 | 2 | C/A | -233.049 | **-507.441** | **-354.655** | **-968.82** | **559.675** | **-835.411** | **-589.402** | 80.671 | 351.228 |
|  | S3_40649278 | 3 | G/A | **-1,010.36** | -243.493 | **-335.716** | 5.793 | **-736.694** | **-573.52** | **-1,046.29** | -252.576 | 270.649 |
|  | S5_187865489 | 5 | G/A | **-321.561** | **766.041** | **306.419** | 300.812 | **403.792** | **535.235** | -47.346 | **1,019.59** | 301.014 |
|  | S5_208598297 | 5 | T/A | **-784.222** | **-614.018** | 167.647 | 81.776 | **-530.549** | -110.651 | **-342.339** | **604.217** | 328.515 |
|  | S7_78837234 | 7 | A/G | -108.01 | -22.961 | **444.776** | **600.985** | **606.038** | **494.434** | **350.312** | **-409.513** | 266.605 |
|  | S8_130875159 | 8 | C/T | -297.649 | **567.288** | **-1,357.18** | 261.541 | -86.704 | 239.236 | -113.595 | **-1.166.80** | 484.225 |
|  | S9_30657359 | 9 | A/C | **1,310.41** | 178.847 | **1,705.87** | -7.226 | **984.175** | 322.924 | **674.859** | 106.305 | 440.704 |
|  | S10_117590335 | 10 | C/G | -336.961 | -241.553 | **-569.125** | -216.724 | **-676.448** | **-2,075.41** | **-669.963** | **-1.276.13** | 446.501 |
|  |  |  |  |  |  |  |  |  |  |  |  |  |
| ASI | S1_12144151 | 1 | G/T | **-0.865** | **-2.169** | 0.374 | -0.408 | **-0.629** | **-1.188** | -0.139 | -0.101 | 0.555 |
|  | S2_25511331 | 2 | G/T | 0.733 | **1.059** | 0.110 | 0.020 | 0.069 | **-2.372** | 0.460 | 0.044 | 0.733 |
|  | S2_48544791 | 2 | G/T | **-0.341** | **-0.341** | **-0.341** | **-0.341** | **-0.341** | **-0.341** | **-0.341** | **-0.341** | 0.169 ^a^ |
|  | S2_235397425 | 2 | G/T | **-0.682** | **-0.656** | 0.239 | -0.184 | **-0.816** | **-0.309** | -0.100 | -0.081 | 0.257 |
|  | S3_186034037 | 3 | T/C | **-1.195** | **-0.859** | 0.033 | -0.054 | -0.101 | 0.059 | 0.328 | 0.057 | 0.370 |
|  | S4_40671352 | 4 | G/A | 0.204 | 0.097 | **1.056** | -0.003 | 0.092 | **0.673** | 0.052 | 0.144 | 0.265 |
|  | S4_155831751 | 4 | T/C | 0.159 | **-0.770** | **-0.532** | **-0.607** | 0.171 | **-1.096** | **-0.417** | -0.252 | 0.310 |
|  | S5_16868689 | 5 | G/T | **-0.262** | 0.172 | **-0.444** | -0.064 | **-0.894** | **-0.281** | -0.099 | -0.086 | 0.226 |
|  | S5_182960002 | 5 | C/T | -0.421 | **-1.766** | **-0.458** | -0.290 | -0.326 | 0.189 | -0.066 | -0.011 | 0.422 |
|  | S6_34603718 | 6 | G/A | **0.627** | **-0.295** | 0.090 | -0.048 | **0.738** | 0.119 | 0.014 | 0.040 | 0.246 |
|  | S6_51074031 | 6 | A/T | **-0.551** | 0.321 | **-1.827** | -0.056 | 0.063 | **-0.670** | 0.099 | -0.398 | 0.480 |
|  | S6_80804844 | 6 | C/T | 0.366 | 0.188 | **-0.904** | -0.054 | 0.179 | **0.405** | 0.159 | **-0.915** | 0.378 |
|  | S7_134329844 | 7 | T/G | **-0.348** | **-0.958** | 0.004 | -0.209 | 0.186 | **-0.425** | -0.153 | **-0.443** | 0.243 |
|  | S7_153536356 | 7 | T/C | -0.057 | **-0.341** | **-0.796** | **-0.494** | 0.037 | -0.016 | 0.051 | -0.083 | 0.215 |
|  | S8_61995434 | 8 | C/T | 0.097 | 0.406 | **1.273** | -0.172 | -0.139 | **-0.455** | -0.170 | **-0.611** | 0.418 |
|  | S9_95035261 | 9 | A/G | -0.184 | 0.105 | 0.185 | 0.062 | -0.156 | **0.773** | **0.376** | -0.063 | 0.224 |
|  | S9_131145152 | 9 | C/A | 0.067 | **-0.622** | 0.086 | 0.215 | -0.121 | **-2.365** | 0.154 | -0.035 | 0.611 |
|  | S10_64393685 | 10 | C/T | **1.037** | 0.212 | 0.165 | 0.037 | **0.345** | **0.325** | 0.165 | 0.026 | 0.229 |
|  | S10_94602385 | 10 | T/A | **0.664** | 0.113 | **-0.587** | 0.211 | **0.298** | 0.170 | **0.322** | 0.124 | 0.248 |
|  |  |  |  |  |  |  |  |  |  |  |  |  |
| FF | S1_9909717 | 1 | T/C | **-0.717** | **-1.672** | **-2.541** | **-1.126** | -0.295 | **-1.093** | -0.300 | -0.539 | 0.543 |
|  | S1_241431165 | 1 | T/A | **-1.872** | -0.394 | -0.042 | 0.190 | **-1.198** | 0.219 | -0.210 | 0.000 | 0.525 |
|  | S1_253967859 | 1 | G/T | 0.082 | -0.077 | **-1.325** | **-0.513** | **0.427** | **- 0.452** | **-0.940** | -0.250 | 0.397 |
|  | S2_216077900 | 2 | G/C | **-2.910** | **-4.505** | -0.932 | **-2.566** | **-2.682** | **-3.191** | **-1.404** | -0.456 | 0.938 |
|  | S3_161608525 | 3 | C/G | **-0.871** | **1.257** | 0.360 | **0.890** | 0.417 | 0.508 | **-1.781** | -0.719 | 0.726 |
|  | S4_150748545 | 4 | A/G | 0.372 | -0.439 | **2.098** | 0.545 | -1.000 | **-3.805** | -0.228 | -0.872 | 1.193 |
|  | S5_3549675 | 5 | T/C | **0.831** | -0.226 | **0.950** | -0.377 | 0.377 | **-1.454** | **-1.105** | 0.246 | 0.613 |
|  | S5_30858109 | 5 | A/G | **-0.402** | **-0.420** | **-1.205** | **-0.674** | -0.276 | **0.513** | 0.192 | **-0.811** | 0.386 |
|  | S7_164722391 | 7 | C/G | 0.149 | **0.631** | 0.178 | -0.303 | 0.199 | 0.303 | -0.278 | **-1.223** | 0.399 |
|  | S8_44997484 | 8 | C/T | -0.550 | **-2.877** | **1.826** | 0.084 | -0.302 | **-1.681** | -0.005 | -0.030 | 0.974 |
|  | S9_141734202 | 9 | C/A | **-0.871** | **-0.444** | **-0.508** | **-1.136** | **-1.817** | **-0.642** | -0.318 | **-0.797** | 0.340 |
|  | S10_4588115 | 10 | C/A | **1.378** | **1.231** | **0.498** | **0.551** | **0.698** | -0.073 | -0.211 | **1.360** | 0.436 |
|  | S10_108841606 | 10 | G/A | 0.189 | -0.373 | -0.730 | **-1.140** | **-0.873** | 0.769 | -0.263 | **-2.998** | 0.790 |
|  | S10_124681296 | 10 | C/A | -0.656 | -1.107 | **-4.900** | -0.013 | 0.542 | -0.120 | -0.596 | 0.638 | 1.251 |
|  |  |  |  |  |  |  |  |  |  |  |  |  |
| MF | S1_33732044 | 1 | C/T | **-0.838** | 0.304 | **-0.491** | **-0.629** | **-1.483** | **-1.095** | **0.565** | -1.088 | 0.500 |
|  | S1_202904615 | 1 | G/C | **0.723** | **0.664** | 0.313 | **0.375** | **1.590** | 0.272 | **0.605** | 0.155 | 0.320 |
|  | S1_256543821 | 1 | T/C | -0.177 | -0.045 | **-1.452** | **-0.855** | 0.117 | -0.193 | **-0.864** | **-1.111** | 0.404 |
|  | S2_60042464 | 2 | C/T | **-0.891** | **-0.891** | **-0.891** | **-0.891** | **-0.891** | **-0.891** | **-0.891** | **-0.891** | 0.277 ^a^ |
|  | S3_21865734 | 3 | T/A | **0.544** | -0.211 | **0.478** | **0.570** | -0.202 | **0.327** | **1.097** | **0.761** | 0.317 |
|  | S4_134650382 | 4 | T/A | **-2.042** | -0.441 | 0.404 | -0.216 | **-0.940** | **-0.734** | **-0.777** | **-0.526** | 0.493 |
|  | S4_202738903 | 4 | T/C | **-0.336** | **-0.730** | **-0.717** | **-0.492** | -0.180 | 0.183 | 0.042 | **-1.069** | 0.299 |
|  | S5_164895579 | 5 | A/G | **-1.172** | **-0.813** | -0.071 | **-0.288** | **-0.726** | **-0.635** | -0.089 | **-0.494** | 0.268 |
|  | S5_213650128 | 5 | T/G | -0.189 | **-1.034** | **-0.309** | **-0.849** | **-0.296** | -0.135 | -0.228 | 0.220 | 0.285 |
|  | S6_34602881 | 6 | G/A | **-0.761** | **-1.654** | **-0.748** | -0.218 | **-0.668** | **-0.709** | **-0.362** | **-0.535** | 0.304 |
|  | S6_48710548 | 6 | C/A | **1.027** | **1.626** | **1.470** | **0.630** | **0.976** | **2.776** | 0.158 | 0.418 | 0.586 |
|  | S6_83128124 | 6 | G/A | 0.149 | 0.122 | 0.576 | 0.850 | 0.901 | 2.721 | -0.020 | 0.517 | 0.618 |
|  | S7_109373491 | 7 | G/A | -0.124 | -0.420 | **-0.578** | 0.228 | -0.274 | **0.720** | **-1.574** | 0.090 | 0.477 |
|  | S8_44997484 | 8 | C/T | **0.881** | **-0.991** | 0.192 | 0.004 | 0.096 | **-1.423** | -0.244 | -0.209 | 0.505 |
|  | S10_114530689 | 10 | G/C | **-0.941** | **-0.941** | **-0.941** | **-0.941** | **-0.941** | **-0.941** | **-0.941** | **-0.941** | 0.348^a^ |
|  | S10_144626525 | 10 | C/T | **-1.365** | **-0.962** | **-1.796** | **-0.959** | **-0.109** | **0.431** | 0.020 | -0.295 | 0.538 |
|  |  |  |  |  |  |  |  |  |  |  |  |  |
|  | S1_201525656 | 1 | A/T | 0.911 | **-5.656** | -4.093 | -3.749 | -2.624 | **-17.446** | 0.845 |  | 4.691 |
| PH | S2_206570246 | 2 | C/T | **17.210** | 0.630 | 2.554 | **16.387** | 6.092 | **-12.096** | **11.279** |  | 7.744 |
|  | S3_11541417 | 3 | A/G | 1.835 | **2.484** | **5.491** | **8.523** | **7.118** | **7.207** | 0.040 |  | 2.434 |
|  | S3_51291956 | 3 | G/A | **-8.351** | **-4.861** | 1.292 | **-2.776** | -1.082 | -1.282 | -1.701 |  | 2.354 |
|  | S4_141637826 | 4 | G/A | -0.453 | -0.468 | **16.577** | 2.324 | 1.191 | 3.823 | -0.542 |  | 4.628 |
|  | S5_21373234 | 5 | G/A | **11.717** | **6.794** | -0.432 | 0.011 | 2.840 | -2.943 | 0.677 |  | 3.793 |
|  | S6_30189713 | 6 | C/A | -2.707 | 3.702 | **14.258** | **6.898** | 2.121 | **11.883** | **19.306** |  | 5.781 |
|  | S6_120791592 | 6 | C/T | -1.301 | -0.934 | **-6.313** | -2.440 | -3.569 | -17.033 | -4.169 |  | 4.208 |
|  | S6_155822086 | 6 | T/G | **4.146** | **6.442** | -0.988 | -0.558 | 0.802 | 7.202 | -4.747 |  | 3.287 |
|  | S7_107518590 | 7 | G/A | **15.370** | **15.370** | **15.370** | **15.370** | **15.370** | **15.370** | **15.370** |  | 6.502^a^ |
|  | S8_144265346 | 8 | C/T | 1.089 | 0.769 | **-7.013** | 0.702 | **7.737** | -3.037 | 0.566 |  | 3.391 |
|  | S9_66796844 | 9 | G/A | **5.693** | **7.404** | **5.430** | **11.524** | 1.138 | **12.048** | **8.087** |  | 2.846 |
|  | S9_137168948 | 9 | C/G | **-9.447** | **-9.447** | **-9.447** | **-9.447** | **-9.447** | **-9.447** | **-9.447** |  | 4.370^a^ |
|  |  |  |  |  |  |  |  |  |  |  |  |  |
|  | S1_223858885 | 1 | G/T | 2.308 | **4.040** | 2.148 | **6.264** | 0.458 | **-4.635** | **4.219** |  | 2.649 |
| EH | S2_32205582 | 2 | A/C | **7.147** | **6.435** | 5.289 | **8.005** | **9.159** | **24.923** | -1.131 |  | 6.012 |
|  | S2_178745233 | 2 | G/A | **-4.569** | **-4.569** | **-4.569** | **-4.569** | **-4.569** | **-4.569** | **-4.569** |  | 2.150^a^ |
|  | S2_218355421 | 2 | T/C | -2.409 | -3.485 | 0.038 | 1.945 | -2.163 | **-11.579** | **7.424** |  | 4.372 |
|  | S3_173518181 | 3 | C/T | **-4.253** | **-11.005** | **-2.683** | **-2.655** | **-5.921** | **-4.717** | **-8.466** |  | 2.336 |
|  | S5_38128373 | 5 | T/C | **9.823** | **6.630** | 1.604 | 0.535 | 1.798 | 0.409 | 1.546 |  | 2.721 |
|  | S5_153236040 | 5 | A/G | 0.255 | **3.498** | **5.267** | **5.370** | **2.178** | **2.140** | **6.674** |  | 1.710 |
|  | S6_164997414 | 6 | G/A | **5.383** | **5.383** | **5.383** | **5.383** | **5.383** | **5.383** | **5.383** |  | 2.790^a^ |
|  | S7_169927644 | 7 | G/A | 0.288 | 0.069 | **-3.544** | -2.099 | **-4.363** | **-11.606** | 0.087 |  | 3.189 |
|  | S8_16773200 | 8 | G/A | **3.412** | 2.540 | 1.533 | 0.072 | **10.531** | **9.691** | 2.192 |  | 3.113 |
|  | S9_98223523 | 9 | C/T | 3.376 | 0.182 | **-11.583** | -0.806 | 2.597 | **5.351** | -2.473 |  | 4.210 |

**^*^**SNP name is represented by letter S followed by the respective chromosome number and the number after the underline character (“_”) is associated with the physical position along the chromosome. ^**^Reference B73 allele is described first, followed by the alternative allele. a: standard error deviation. PH and EH traits were not evaluated at environment T11WW.

**Table S7.** Significant main and environment-specific associations detected through the MEML association mapping model for the Flint mapping group. Bold values represent significant effects considering a threshold of $2\times\vee\overline{se}\vee$, being $\overline{se}$ the mean residual standard error). Evaluated traits were: grain yield (GY), anthesis-silking interval (ASI), female flowering time (FF), male flowering time (MF), plant heigh (PH) and ear height (EH). Chr: chromosome. Environment: J10WS, J11WS, T10WS, T11WS, J10WW, J11WW, T10WW, and T11WW.

| Trait | SNP^*^ | Chr | Allele^**^ | J10WS | J11WS | T10WS | T11WS | J10WW | J11WW | T10WW | T11WW | $2\overline{se}$ |
| --- | --- | --- | --- | --- | --- | --- | --- | --- | --- | --- | --- | --- |
| GY | S1_6527154 | 1 | C/A | **-285.08** | -143.674 | -145.806 | 103.586 | **-913.347** | -27.853 | -**269.889** | -111.655 | 215.806 |
|  | S1_22650134 | 1 | T/A | 3.48 | 129.019 | **456.386** | **505.469** | **803.627** | **542.75** | **553.986** | **-259.74** | 247.876 |
|  | S1_148462322 | 1 | A/G | **531.82** | 247.064 | 58.719 | 119.453 | **1,082.17** | 48.585 | -326.59 | **859.648** | 329.726 |
|  | S1_280448428 | 1 | C/T | **446.40** | **446.40** | **446.40** | **446.40** | **446.40** | **446.40** | **446.40** | **446.40** | 162.760^a^ |
|  | S2_182837718 | 2 | A/G | **857.70** | **857.70** | **857.70** | **857.70** | **857.70** | **857.70** | **857.70** | **857.70** | 338.600 ^a^ |
|  | S4_128260623 | 4 | G/A | **619.463** | -16.857 | 170.476 | **339.703** | **416.75** | -104.335 | -32.334 | **790.093** | 230.322 |
|  | S4_186220775 | 4 | A/C | -221.085 | **513.388** | 49.237 | 156.068 | **-501.446** | 248.909 | 71.144 | **733.639** | 274.921 |
|  | S5_69003739 | 5 | A/G | 155.058 | **-390.117** | 121.921 | -168.993 | **363.551** | **-536.314** | **-281.244** | **285.765** | 235.26 |
|  | S5_212696866 | 5 | A/G | 15.407 | -390.463 | **1,340.41** | 546.77 | -309.29 | **-1,910.46** | -328.649 | **-1,648.00** | 752.003 |
|  | S6_6899291 | 6 | C/G | -316.722 | -8.326 | **393.392** | **583.869** | 98.857 | **-705.624** | -128.576 | **-1,075.89** | 388.208 |
|  | S6_144171977 | 6 | G/A | -62.084 | -102.575 | **-982.632** | **-352.225** | -182.67 | -37.886 | 88.671 | **794.679** | 347.509 |
|  | S7_74540732 | 7 | A/G | -27.348 | -142.097 | **-324.176** | **-784.90** | -63.886 | -101.962 | -157.87 | 26.07 | 183.544 |
|  | S7_143580075 | 7 | A/G | 177.281 | -277.297 | **-547.881** | 215.864 | **-417.452** | **-444.748** | **-1,161.88** | **-1,462.05** | 413.669 |
|  |  |  |  |  |  |  |  |  |  |  |  |  |
| ASI | S1_56413930 | 1 | G/A | -0.324 | -0.221 | **-1.674** | **-0.860** | 0.069 | -0.319 | -0.228 | -0.152 | 0.393 |
|  | S1_274549233 | 1 | C/G | **0.691** | **-1.924** | -0.288 | 0.173 | 0.443 | -0.064 | 0.094 | -0.219 | 0.561 |
|  | S1_288545551 | 1 | C/A | **-0.993** | **-2.498** | 0.318 | -0.449 | **-1.003** | **-0.706** | 0.133 | 0.016 | 0.638 |
|  | S2_4700349 | 2 | T/G | 0.040 | 0.173 | **-2.331** | 0.097 | -0.524 | **-0.763** | **-0.710** | -0.195 | 0.577 |
|  | S3_58908138 | 3 | T/C | 0.088 | 0.040 | **0.388** | **0.668** | -0.096 | -0.051 | 0.094 | 0.137 | 0.178 |
|  | S3_150540151 | 3 | C/T | **0.882** | **0.835** | **-0.492** | -0.044 | **0.429** | **0.835** | 0.019 | 0.058 | 0.359 |
|  | S3_184323470 | 3 | T/A | -0.027 | 0.137 | **-1.939** | -0.410 | -0.092 | 0.079 | -0.128 | -0.159 | 0.478 |
|  | S4_129977060 | 4 | C/A | -0.366 | **-1.802** | **-3.778** | 0.225 | 0.022 | 0.884 | -0.132 | 0.363 | 1.069 |
|  | S5_185078886 | 5 | C/T | **-1.809** | **-1.239** | **0.718** | **-0.585** | **-0.603** | -0.470 | -0.151 | 0.094 | 0.550 |
|  | S6_48712597 | 6 | A/G | **-0.628** | -0.279 | **-1.304** | **-0.401** | **-0.905** | **-0.874** | -0.205 | -0.211 | 0.282 |
|  | S7_113024953 | 7 | C/T | -0.158 | **0.284** | **-0.297** | **-0.433** | **-0.278** | **-0.958** | -0.035 | -0.135 | 0.252 |
|  | S7_175270831 | 7 | G/C | -0.049 | -0.416 | **-3.166** | 0.196 | 0.139 | **-0.815** | -0.071 | 0.075 | 0.794 |
|  | S8_935523 | 8 | G/C | 0.016 | **-1.032** | **-0.851** | -0.071 | **-0.350** | 0.243 | 0.092 | -0.082 | 0.324 |
|  | S8_96277038 | 8 | G/A | -0.231 | **-0.897** | **-0.719** | -0.115 | **-0.618** | **-0.689** | 0.034 | **-1.024** | 0.271 |
|  | S9_138961140 | 9 | C/G | **-0.950** | 0.063 | **-0.783** | **0.382** | **-0.676** | **-0.854** | -0.201 | -0.165 | 0.344 |
|  | S10_15149198 | 10 | G/A | -0.054 | -0.063 | **0.720** | **-0.381** | 0.097 | **0.346** | -0.131 | **0.340** | 0.244 |
|  |  |  |  |  |  |  |  |  |  |  |  |  |
| FF | S1_8333264 | 1 | G/T | 0.134 | -0.346 | -**4.389** | 0.160 | -0.591 | -0.777 | -0.149 | -0.608 | 1.048 |
|  | S1_30572798 | 1 | A/C | **1.140** | **-0.590** | -0.268 | -0.325 | **0.532** | -0.048 | -0.020 | 0.223 | 0.388 |
|  | S1_82806970 | 1 | T/A | -0.150 | **1.135** | **2.048** | **0.845** | 0.284 | **1.805** | **0.748** | 0.482 | 0.526 |
|  | S2_30149856 | 2 | C/T | 0.280 | **-0.909** | 0.276 | **-0.979** | 0.003 | -0.048 | **-1.023** | **-1.851** | 0.545 |
|  | S2_156963387 | 2 | G/A | **-0.424** | 0.040 | **-1.048** | 0.197 | **-0.491** | -0.370 | **-1.303** | -0.048 | 0.370 |
|  | S2_178545968 | 2 | G/A | -0.802 | **-3.528** | 0.544 | **-1.362** | **-1.643** | -0.150 | 0.680 | **-0.959** | 0.957 |
|  | S4_16367462 | 4 | G/A | -0.050 | -0.116 | **0.738** | **-2.061** | 0.211 | **0.725** | -0.130 | -0.105 | 0.617 |
|  | S4_47482272 | 4 | C/T | **-0.650** | -0.088 | **-2.157** | **-0.991** | -0.505 | -0.107 | 0.210 | -0.004 | 0.539 |
|  | S4_73211479 | 4 | G/A | 0.086 | **1.391** | 0.186 | **-1.442** | **1.006** | **-1.013** | -0.362 | 0.078 | 0.667 |
|  | S4_160006472 | 4 | C/T | -0.471 | **-1.551** | **-3.215** | **-0.837** | **-1.940** | **-1.290** | -0.219 | **-2.091** | 0.689 |
|  | S5_3659988 | 5 | G/A | -0.106 | 0.540 | **1.181** | **1.132** | **1.000** | 0.050 | **-0.727** | **-1.433** | 0.667 |
|  | S5_32942014 | 5 | A/G | -0.588 | **-4.567** | **-5.002** | -0.618 | **-1.654** | -1.333 | 0.474 | -1.023 | 1.383 |
|  | S5_214163354 | 5 | G/T | **-1.091** | **-1.567** | **-1.975** | **-2.087** | **-0.890** | **-1.300** | -0.179 | -0.383 | 0.489 |
|  | S6_28097395 | 6 | G/C | **-1.384** | **0.530** | **0.877** | -0.159 | **-0.690** | -0.101 | -0.322 | 0.430 | 0.513 |
|  | S6_117326542 | 6 | C/T | 0.090 | **0.495** | -0.187 | **0.380** | **0.397** | 0.138 | **0.250** | **0.967** | 0.240 |
|  | S8_1449352 | 8 | C/A | **-0.895** | **-0.730** | **-0.820** | **-1.168** | -0.351 | **-0.831** | **-2.476** | -0.077 | 0.505 |
|  | S8_172969010 | 8 | A/G | **-0.680** | -0.397 | **-0.732** | **0.702** | -0.506 | **-1.331** | **-2.099** | -0.415 | 0.569 |
|  | S10_78814808 | 10 | T/C | **-0.904** | -0.155 | **-2.265** | **-0.840** | **-0.677** | **-1.318** | -0.257 | -**1.882** | 0.526 |
|  |  |  |  |  |  |  |  |  |  |  |  |  |
| MF | S1_42930560 | 1 | G/C | **-0.845** | -0.230 | **0.424** | **-0.532** | **-0.514** | **-0.595** | **-0.321** | -0.076 | 0.274 |
|  | S2_30149856 | 2 | C/T | 0.300 | 0.401 | **0.850** | -0.391 | -0.462 | 0.334 | **-0.558** | **-1.666** | 0.558 |
|  | S2_179660213 | 2 | T/C | **0.316** | 0.008 | **-0.949** | **-0.817** | -0.199 | **-0.438** | -0.182 | **-0.668** | 0.305 |
|  | S3_35930512 | 3 | A/G | **0.429** | **1.157** | 0.204 | **0.446** | **0.649** | **0.746** | -0.214 | **0.343** | 0.285 |
|  | S3_58592255 | 3 | C/A | -0.230 | **-1.653** | **-1.690** | **-0.686** | **-1.250** | **-0.625** | **0.591** | -0.083 | 0.565 |
|  | S4_160006472 | 4 | C/T | **-0.684** | -0.020 | 0.023 | **-0.638** | **-1.272** | **-1.133** | -0.117 | -**1.757** | 0.460 |
|  | S5_61021766 | 5 | T/G | 0.014 | -0.469 | -0.279 | -0.555 | 0.085 | **-2.800** | **-0.856** | 0.151 | 0.678 |
|  | S5_155565583 | 5 | G/C | 0.331 | -0.268 | **-1.275** | 0.002 | -0.044 | -0.356 | -0.284 | **-1.399** | 0.432 |
|  | S5_168457912 | 5 | T/C | **0.705** | **1.742** | **0.565** | -0.373 | **1.129** | **1.545** | -0.411 | 0.452 | 0.563 |
|  | S5_206328630 | 5 | C/T | **0.569** | **0.766** | **1.179** | 0.197 | 0.215 | **1.170** | **0.384** | 0.313 | 0.284 |
|  | S6_70762019 | 6 | A/G | -0.149 | **-1.087** | -0.129 | 0.274 | -0.160 | **-0.496** | -0.210 | 0.019 | 0.286 |
|  | S7_157513376 | 7 | C/G | -0.144 | -0.017 | 0.225 | **-0.624** | 0.148 | **-0.526** | **-1.980** | -0.066 | 0.505 |
|  | S8_62543145 | 8 | C/T | 0.487 | -0.083 | **-2.151** | -0.230 | 0.000 | -0.111 | 0.149 | 0.113 | 0.570 |
|  | S8_150115687 | 8 | C/A | -0.263 | -0.211 | **-1.820** | **-0.940** | 0.010 | **-0.716** | **-0.487** | **-1.001** | 0.412 |
|  | S9_113083861 | 9 | A/C | **0.545** | **0.239** | **-0.339** | **0.288** | **0.743** | **0.315** | **0.323** | 0.073 | 0.226 |
|  | S9_144785518 | 9 | A/C | -0.110 | 0.410 | **-2.866** | -0.206 | 0.249 | 0.413 | -0.134 | -0.439 | 0.755 |
|  | S10_4614029 | 10 | T/A | **-1.401** | **-1.222** | **-0.683** | **-0.401** | **-0.598** | -0.106 | **-0.835** | **-0.637** | 0.296 |
|  | S10_97468985 | 10 | C/T | **-0.592** | -0.097 | **-0.381** | 0.031 | **-0.526** | **0.313** | -0.167 | **-0.305** | 0.212 |
|  |  |  |  |  |  |  |  |  |  |  |  |  |
| PH | S1_211513353 | 1 | G/T | -3.011 | -2.532 | **7.785** | 0.573 | **-6.572** | **-8.868** | -0.695 |  | 4.063 |
|  | S1_270739240 | 1 | A/T | 2.611 | 2.698 | **-3.202** | **5.362** | **7.988** | 0.003 | 1.008 |  | 2.746 |
|  | S2_9507977 | 2 | T/C | **-6.012** | **-8.244** | 0.235 | **-12.671** | **-4.861** | **-5.663** | -0.650 |  | 3.324 |
|  | S2_197180515 | 2 | G/A | **8.862** | **4.747** | **6.069** | -4.112 | **5.704** | **-7.558** | -2.856 |  | 4.739 |
|  | S2_218308899 | 2 | T/C | -2.514 | -2.376 | **-7.420** | 2.188 | **7.908** | **11.197** | **12.981** |  | 5.850 |
|  | S3_214903876 | 3 | C/A | **-6.836** | 0.981 | **-5.147** | 0.496 | -1.697 | 1.087 | -1.855 |  | 2.348 |
|  | S4_234175303 | 4 | G/T | 2.110 | **4.520** | **16.916** | **10.548** | **8.536** | 0.811 | **10.051** |  | 4.232 |
|  | S5_77783739 | 5 | C/A | -3.384 | 1.867 | **-8.573** | -3.710 | -0.810 | **-13.885** | -2.334 |  | 3.968 |
|  | S5_174182052 | 5 | G/T | 0.873 | **7.825** | -3.166 | -1.311 | 1.598 | **4.697** | **-3.767** |  | 3.178 |
|  | S6_154505013 | 6 | C/G | **4.863** | 2.910 | **-13.577** | -2.135 | 1.902 | 3.223 | -4.202 |  | 4.837 |
|  | S7_67197741 | 7 | C/T | -2.339 | 4.280 | -3.498 | **13.312** | 2.206 | **4.547** | **7.144** |  | 4.318 |
|  | S7_150046031 | 7 | T/A | **-5.624** | **-3.002** | **5.597** | **3.071** | 1.161 | -1.085 | 0.118 |  | 2.827 |
|  | S8_72256195 | 8 | C/T | **-9.085** | **-9.085** | **-9.085** | **-9.085** | **-9.085** | **-9.085** | **-9.085** |  | 3.374^a^ |
|  | S9_14465449 | 9 | G/C | **7.775** | **7.457** | **12.890** | **4.961** | -0.521 | **4.147** | 2.641 |  | 3.238 |
|  | S10_100044876 | 10 | C/T | 0.856 | **5.904** | **10.691** | **16.546** | 3.610 | **8.007** | **-6.369** |  | 5.532 |
|  |  |  |  |  |  |  |  |  |  |  |  |  |
| EH | S1_53171490 | 1 | T/C | **-4.817** | **-4.817** | **-4.817** | **-4.817** | **-4.817** | **-4.817** | **-4.817** |  | 2.450^a^ |
|  | S1_256053397 | 1 | A/G | **3.381** | -2.050 | -**6.041** | -5.021 | -2.564 | **-5.533** | **-7.826** |  | 2.790 |
|  | S2_214431087 | 2 | A/G | **-5.571** | **-5.571** | **-5.571** | **-5.571** | **-5.571** | **-5.571** | **-5.571** |  | 2.084^a^ |
|  | S2_233784862 | 2 | G/T | **-5.590** | **-5.326** | -3.148 | 1.386 | -1.409 | **-12.509** | -3.207 |  | 3.285 |
|  | S3_3550591 | 3 | G/A | 1.849 | 2.568 | **9.084** | 7.936 | -1.223 | **-4.602** | **8.694** |  | 4.016 |
|  | S3_210507376 | 3 | C/T | **-6.312** | **-6.312** | **-6.312** | **-6.312** | **-6.312** | **-6.312** | **-6.312** |  | 2.750^a^ |
|  | S4_185816529 | 4 | G/C | -0.894 | -2.034 | **6.951** | 4.762 | 1.932 | **7.268** | **6.657** |  | 2.928 |
|  | S5_166688086 | 5 | C/T | 1.471 | -0.448 | **4.396** | 2.136 | 0.499 | -0.283 | **9.936** |  | 2.769 |
|  | S5_182568689 | 5 | G/C | -6.520 | 2.665 | **4.873** | -1.041 | **-3.240** | 0.216 | 1.328 |  | 2.867 |
|  | S7_174850812 | 7 | G/T | -0.637 | -2.116 | **-5.777** | -11.136 | **-6.952** | -0.551 | 0.813 |  | 3.271 |
|  | S8_853385 | 8 | T/A | -2.742 | -0.703 | 3.733 | 0.843 | 2.842 | -**11.991** | 0.249 |  | 3.976 |
|  | S9_14465449 | 9 | G/C | **5.764** | **7.973** | **5.997** | 3.601 | 1.322 | -1.285 | 0.274 |  | 2.569 |
|  | S9_80631489 | 9 | G/T | -2.648 | **4.336** | **4.283** | 11.435 | 1.534 | **5.222** | -1.428 |  | 3.556 |
|  | S9_135712464 | 9 | G/A | -1.797 | -0.633 | **-3.736** | **-3.665** | **-5.384** | **-8.136** | **-8.929** |  | 2.331 |

**^*^**SNP name is represented by letter S followed by the respective chromosome number and the number after the underline character (“_”) is associated with the physical position along the chromosome. ^**^Reference allele is described first, followed by the alternative allele. a: standard error deviation. PH and EH traits were not evaluated at environment T11WW.

**Table S8: SNPs associated with drought-related traits that were integrated with the PlantAnnot2 platform**

| Associated SNP | Trait | Effect | Candidate Gene | Start (bp) | Stop (bp) | Predicted ID | Expression Data at PlantAnnot2 Platform | | | | | |
| --- | --- | --- | --- | --- | --- | --- | --- | --- | --- | --- | --- | --- |
|  |  |  |  |  |  |  | Assays | Stress | Biological material | TPM^*^ | Cluster^#^ | Number mRNAs^#^ |
| S2_182837718 | GY | Main | GRMZM2G159125 | 182,836,551 | 182,840,311 | Phospholipase D alpha 1-like | SRR2129983 | Drought | Leaf | 0.05 | zma_coexpr_cluster_0005 | 3,161 |
|  |  |  |  |  |  |  | SRR2106186, SRR2106198, SRR2106202 | Salinity | Leaf | 0.13 to 0.41 |  |  |
|  |  |  |  |  |  |  | SRR3105596, SRR3105597 | Cold | Seedling | 0.05 |  |  |
| S3_210507376 | EH | Main | GRMZM2G402088 | 210,501,079 | 210,512,481 | PHD-like zinc-binding domain (zf-HC5HC2H) | SRR2129983, SRR2144414, SRR2144422, SRR2144430, SRR2144438 | Drought | Leaf | 1.08 to 13.80 | zma_coexpr_cluster_0006 | 2,926 |
|  |  |  |  |  |  |  | SRR2106186, SRR2106198, SRR2106202 | Salinity | Leaf | 7.68 to 9.45 |  |  |
|  |  |  |  |  |  |  | SRR3105596, SRR3105597 | Cold | Seedling | 4.52 to 5.30 |  |  |
| S2_235397425 | ASI | QEI | GRMZM5G805105 | 235,395,404 | 235,397,906 | Zinc-finger of C2H2 | SRR2129983, SRR2144414, SRR2144422, SRR2144430, SRR2144438 | Drought | Leaf | 1.80 to 11.83 | zma_coexpr_cluster_0006 | 2,926 |
|  |  |  |  |  |  |  | SRR2106186, SRR2106198, SRR2106202 | Salinity | Leaf | 5.81 to 6.57 |  |  |
|  |  |  |  |  |  |  | SRR3105596, SRR3105597 | Cold | Seedling | 2.42 to 2.99 |  |  |
| S9_95035261 | ASI | QEI | GRMZM2G087806 (ZmTIP1) | 95,033,444 | 95,044,390 | S-acyltransferase | SRR2129983, SRR2144414, SRR2144422, SRR2144430, SRR2144438 | Drought | Leaf | 5.19 to 26.94 | zma_coexpr_cluster_0003 | 4,310 |
|  |  |  |  |  |  |  | SRR2106186, SRR2106198, SRR2106202 | Salinity | Leaf | 27.39 to 32.73 |  |  |
|  |  |  |  |  |  |  | SRR3105596, SRR3105597 | Cold | Seedling | 15.38 to 18.21 |  |  |

^*^ TPM: normalized score of transcripts per million; ^#^ Coexpression clusters followed by the number of mRNA within each cluster

**Figure S1.** SNP percentage relative to the minor allele frequency before data filtering procedure, considering the genotypic information of the association mapping panel for drought tolerance from the Embrapa Maize and Sorghum maize breeding program.

**Figure S2.** SNP density along each of the ten maize chromosomes after filtering.

**Figure S3.** LD mean ($r^{2}$) of SNPs estimated in window size mean of 1 Mb along the ten chromosomes of maize. Centromeric regions are indicate by black points along each chromosome. Physical positions are represented in megabase (Mb).

**Figure S4.** Linkage disequilibrium decay between all pairs of SNP markers along the ten chromosomes of maize. LD mean ($r^{2}$) is a function of marker distances. kb: kilobase; Mb (megabase).
